# Neutrophils and neutrophil extracellular traps in ischaemia–reperfusion injury: pathophysiological roles and therapeutic potential

**DOI:** 10.1093/burnst/tkag022

**Published:** 2026-03-16

**Authors:** Yiqiong Zhang, Chaofu Li, Qiuyan Jiang, Yukun Yang, Yingying Jiang, Lin Chen, Xiang Wei, Jun Xiao, Chuanwei Li

**Affiliations:** Department of Cardiology, Chongqing Key Laboratory of Emergency Medicine, Chongqing University Central Hospital (Chongqing Emergency Medical Center), School of Medicine, Chongqing University, No. 1 Jiankang Road, Yuzhong District, 400014 Chongqing, China; Department of Cardiology, Chongqing Key Laboratory of Emergency Medicine, Chongqing University Central Hospital (Chongqing Emergency Medical Center), School of Medicine, Chongqing University, No. 1 Jiankang Road, Yuzhong District, 400014 Chongqing, China; Department of Cardiology, Chongqing Key Laboratory of Emergency Medicine, Chongqing University Central Hospital (Chongqing Emergency Medical Center), School of Medicine, Chongqing University, No. 1 Jiankang Road, Yuzhong District, 400014 Chongqing, China; Department of Neurology, University Hospital Essen, University of Duisburg-Essen, Hufelandstraße 55, 45147 Essen, North Rhine-Westphalia, Germany; Department of Cardiology, Chongqing Key Laboratory of Emergency Medicine, Chongqing University Central Hospital (Chongqing Emergency Medical Center), School of Medicine, Chongqing University, No. 1 Jiankang Road, Yuzhong District, 400014 Chongqing, China; Department of Cardiology, Chongqing Key Laboratory of Emergency Medicine, Chongqing University Central Hospital (Chongqing Emergency Medical Center), School of Medicine, Chongqing University, No. 1 Jiankang Road, Yuzhong District, 400014 Chongqing, China; Institute of Cardiovascular Physiology and Pathophysiology, Biomedical Center, Ludwig-Maximilians-Universität München, Großhaderner Straße 9, 82152 Planegg-Martinsried, Germany; Department of Cardiology, Chongqing Key Laboratory of Emergency Medicine, Chongqing University Central Hospital (Chongqing Emergency Medical Center), School of Medicine, Chongqing University, No. 1 Jiankang Road, Yuzhong District, 400014 Chongqing, China; Department of Cardiology, Chongqing Key Laboratory of Emergency Medicine, Chongqing University Central Hospital (Chongqing Emergency Medical Center), School of Medicine, Chongqing University, No. 1 Jiankang Road, Yuzhong District, 400014 Chongqing, China

**Keywords:** Ischaemia–reperfusion injury, Neutrophils, Neutrophil extracellular traps, Inflammatory response

## Abstract

Ischaemia–reperfusion injury (IRI) is a fundamental pathological process underlying acute and chronic damage associated with myocardial infarction, ischaemic stroke, and solid organ transplantation. Although timely reperfusion is indispensable for tissue salvage, it paradoxically promotes maladaptive immune activation and oxidative stress, which aggravate microvascular dysfunction and organ failure. Accumulating evidence indicates that sterile inflammation, endothelial injury, and immunothrombosis are the central drivers of IRI progression. Among innate immune effectors, neutrophils act as first responders that integrate chemotactic signalling, adhesion cascades, and metabolic rewiring. Upon activation, neutrophils release damage-associated molecular patterns and form neutrophil extracellular traps (NETs), which amplify inflammation, promote coagulation, and disrupt tissue repair across organs. However, the organ-specific roles, temporal dynamics, and translational relevance of neutrophils and NETs in IRI remain incompletely understood. In this review, we systematically dissect the neutrophil- and NET-mediated mechanisms involved in IRI across the heart, brain, kidney, liver, and transplanted organs, with a particular emphasis on endothelial crosstalk, immunothrombosis, and metabolic regulation. We further summarize emerging NET-associated biomarkers—including cell-free DNA and myeloperoxidase–DNA complexes—for IRI diagnosis and prognosis. Finally, we evaluate therapeutic strategies targeting neutrophil recruitment, immune metabolism, and NET clearance, highlighting challenges for clinical translation. In summary, this review provides a mechanistic and translational framework for targeting neutrophils and NETs in precision therapies for IRI.

## Graphical Abstract

**Graphical Abstract:**
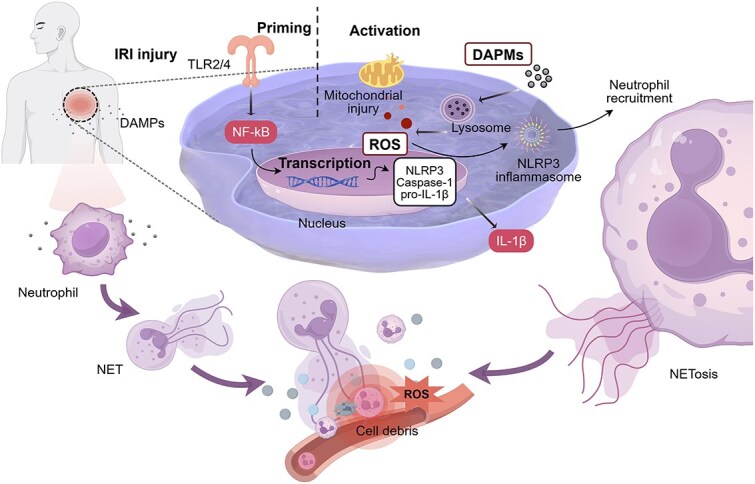

**Highlights**

- Systematically delineates the pivotal role of the neutrophil–NET axis in mediating ischaemia–reperfusion injury.
- Introduces neutrophil heterogeneity and the molecular mechanisms driving NET formation.
- Summarizes current NET-targeted therapeutic strategies, spanning mechanistic insights to clinically approved interventions.

## Background

Ischaemia–reperfusion injury (IRI) is a shared pathological process underlying several high-mortality conditions, including myocardial infarction, ischaemic stroke, and graft dysfunction after organ transplantation. Although the rapid restoration of blood flow is indispensable for salvaging ischaemic tissue, the reperfusion phase itself can initiate secondary injury. Intense inflammatory responses, oxidative stress, and microcirculatory dysfunction frequently accompany reperfusion, leading to tissue damage that extends beyond the original ischaemic region. Consequently, secondary injury may exceed the primary ischaemic insult, resulting in an enlarged infarct size, impaired neurological recovery, or primary graft nonfunction, ultimately limiting the therapeutic benefit of current reperfusion-based interventions.

Despite substantial differences in initiating triggers and clinical manifestations across organs, the core pathological features of IRI are markedly conserved; these include injury to vascular endothelial and parenchymal cells, excessive innate immune system activation, and microvasculature disruption. Within this shared pathological context, neutrophils are among the earliest immune cells to be recruited to sites of injury and play a central role in amplifying tissue damage through degranulation, reactive oxygen species (ROS) generation, and the release of pro-inflammatory mediators. Advances in the understanding of neutrophil effector functions have drawn increasing attention to neutrophil extracellular traps (NETs), which are web-like structures composed of decondensed chromatin and granular proteins that are recognized as important contributors to inflammatory amplification, immune dysregulation, and microvascular injury.

Accumulating clinical and experimental evidence suggests that NETs not only are byproducts of inflammation during IRI but also actively participate in sustaining tissue injury. NETs can exert direct cytotoxic effects, compromise endothelial barrier integrity, promote microthrombus formation, and reinforce inflammatory feedback loops through the release of damage-associated molecular patterns (DAMPs). In patients with acute myocardial infarction, circulating NET-associated biomarkers are correlated with infarct size and adverse outcomes. Experimental models of ischaemic stroke have demonstrated that NET deposition obstructs cerebral microvessels and aggravates blood–brain barrier disruption. Similarly, in organ transplantation—particularly lung transplantation—perioperative elevations in NET levels are closely associated with the development of primary graft dysfunction. Together, these findings highlight the recurring involvement of NETs across distinct IRI settings [1].

Despite this growing body of evidence, current knowledge remains fragmented across disease-specific models, and direct comparisons across organs and temporal stages of IRI are limited. Moreover, the processes governing NET formation and clearance appear to be, at least in part, amenable to therapeutic intervention, raising interest in NETs as potential treatment targets. However, the mechanisms that regulate NET dynamics at different phases of IRI, their upstream signalling networks, and their organ-specific pathological roles remain incompletely defined. Clarifying these issues is essential for the development of precise, time-sensitive therapeutic strategies [2].

**Figure 1:**
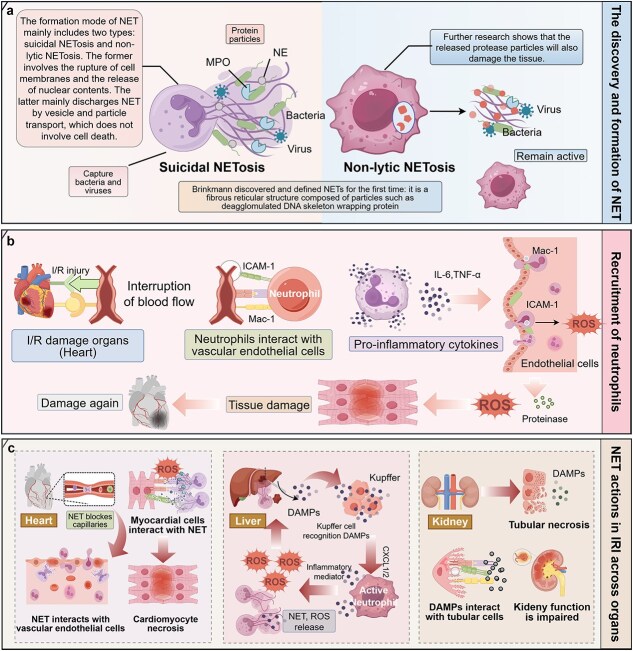
NETs and their role in IRI. (**a**) Discovery and formation of NETs: NETs are web-like structures composed of decondensed chromatin and granular proteins. They form via two main mechanisms: suicidal NETosis, involving cell membrane rupture and nuclear content release, and non-lytic NETosis, where NET components are secreted through vesicles without cell death. Initially described as antimicrobial, NETs are now known to cause tissue injury in sterile inflammation due to toxic components such as histones and proteases. (**b**) Recruitment of neutrophils to injured tissues: During IRI, the cessation and restoration of blood flow trigger pro-inflammatory cytokines (e.g. IL-6, TNF-α) and DAMPs, which activate neutrophils and recruit them to injury sites. Adhesion is mediated by Mac-1 on neutrophils binding to ICAM-1 on endothelial cells, promoting transendothelial migration. Activated neutrophils release ROS, proteases, and NETs, which exacerbate endothelial damage and intensify inflammation. (**c**) Organ-specific actions of NETs in IRI: In the heart, NETs interact with endothelium and cardiomyocytes, promoting dysfunction and necrosis. In the liver, Kupffer cells sense DAMPs and activate neutrophils, which release NETs and ROS, worsening injury. In the kidney, DAMPs stimulate tubular cells to promote NET formation, leading to tubular necrosis and impaired renal function. These processes collectively highlight NET-mediated damage across organs during IRI. *NET*s neutrophil extracellular traps, *IRI* ischaemia–reperfusion injury, *ROS* reactive oxygen species, *DAMPs* damage-associated molecular patterns, *IL-6* interleukin-6, *TNF-α* tumour necrosis factor-alpha, *ICAM-1* intercellular adhesion molecule-1, *Mac-1* integrin macrophage-1 antigen

In light of this background, the present review systematically examines the role of NETs in IRI. We first outline the principal triggers and molecular regulatory pathways involved in NET formation under IRI conditions. We then integrate evidence from diverse organ models to compare the shared and context-dependent roles of NETs in tissue injury, microvascular occlusion, and inflammatory propagation. Finally, we summarize current therapeutic approaches targeting NET formation, stability, and clearance and discuss key challenges related to intervention timing and safety in clinical translation. Through this structured framework, this review aims to provide a coherent view of NET-driven immunopathology in IRI and to inform future therapeutic development [3] (Figure 1).

## Review

### Neutrophil heterogeneity

Neutrophils constitute the most abundant granulocyte population in peripheral blood. Their development originates from haematopoietic stem cells and proceeds through common myeloid progenitors, granulocyte–monocyte progenitors, and cells in successive stages of granulocytic differentiation, including myeloblasts, promyelocytes, myelocytes, and metamyelocytes, ultimately leading to the generation of banded and fully mature neutrophils [4]. Throughout this maturation process, neutrophils progressively acquire diverse immune effector functions, including the capacity to form NETs. For a long time, neutrophils were regarded as a homogeneous population of terminally differentiated cells. However, advances in single-cell transcriptomics, spatial transcriptomics, and mass cytometry have fundamentally revised this view by revealing substantial heterogeneity in neutrophil phenotypes, functional properties, and developmental states [5]. More recently, single-cell RNA sequencing (scRNA-seq) studies have further clarified the molecular basis of this heterogeneity by identifying subtype-specific transcription factor networks and epigenetic regulatory programs, demonstrating that transitions between distinct neutrophil states are governed by tightly controlled gene regulatory modules [6].

Within this framework of developmental and functional heterogeneity, age-associated neutrophil states have emerged as a representative example of dynamic neutrophil plasticity [7]. A study has demonstrated clear distinctions between aged neutrophils and newly released neutrophils from the bone marrow. Aged neutrophils are characterized by the loss of cluster of differentiation 62L (CD62L) expression and the upregulation of C-X-C chemokine receptor type 4 (CXCR4) and cluster of differentiation 11b (CD11b) expression, a phenotypic shift that promotes the homing of neutrophils back to the bone marrow for clearance. In addition to this trafficking behaviour, aged neutrophils exhibit enhanced integrin activation and an increased capacity to form NETs, thereby sustaining inflammatory responses during the early phase of reperfusion [8]. Following cell death, however, neutrophils can switch to an anti-inflammatory role by generating a distinct population of large senescent neutrophil-derived vesicles, termed large ageing neutrophil-derived vesicles (LAND-Vs). These vesicles are enriched in the surface molecules cluster of differentiation 47 (CD47) and cluster of differentiation 55 (CD55). CD47 functions as a canonical ‘don’t eat me’ signal, protecting LAND-Vs from phagocytic clearance, whereas CD55 mediates anti-inflammatory effects by inhibiting complement activation and modulating immune responses, thereby limiting tissue injury associated with excessive neutrophil recruitment [9]. Consistent with these functions, the administration of purified LAND-Vs has been shown to significantly improve survival in a murine model of *Staphylococcus aureus* pneumonia. Moreover, in patients with COVID-19, plasma levels of CD55^+^CD47^+^ LAND-Vs are positively correlated with favourable clinical outcomes, highlighting their cross-species conservation and potential therapeutic relevance.

Beyond age-related states, neutrophil heterogeneity is further shaped by functional specialization defined through surface receptor activity and context-dependent gene expression. Additional neutrophil subsets have been identified on the basis of Fc receptor functional diversity as well as the expression of C-C motif chemokine ligand 3 (CCL3) and Ym-1. Using IgG-coated human erythrocyte rosette formation assays, neutrophils can be stratified into two subsets according to Fc receptor functionality, among which ~20% exhibit reduced Fc receptor activity and may correspond to an anti-inflammatory or regulatory phenotype. In the early phase of IRI, neutrophil heterogeneity is characterized by the differential expression of CCL3 and Ym-1, with myeloid-related protein 1 (Ym-1)–high neutrophils displaying a reparative phenotype [10]. Elevated Ym-1 expression is closely associated with tissue repair and angiogenesis and has cardioprotective effects through the modulation of macrophage polarization. However, mechanistic evidence supporting this subset is derived predominantly from murine models. Corresponding homologous neutrophil populations in humans, as well as relevant prognostic data, remain insufficiently characterized. Therefore, further validation in large-animal models and early-phase clinical studies is needed to assess the therapeutic efficacy and safety of homologous genes or related neutrophil subsets before their translational potential can be fully established [10].

In parallel with functional and transcriptional heterogeneity, surface marker–defined neutrophil subsets provide additional insight into the context-dependent roles of neutrophils in IRI. CD177 is a cell surface protein that is heterogeneously expressed among neutrophils [11]. In humans, the distribution of CD177 is bimodal, with ~40%–60% of neutrophils expressing CD177 and 3%–5% of individuals completely lacking CD177 expression. Accumulating evidence suggests that neutrophil CD177 is involved in inflammatory tissue injury following IRI [12]. Single-cell analyses have demonstrated the enrichment of CD177 expression in IRI-affected tissues, where CD177 promotes inflammatory damage by upregulating mitochondrial complex I expression, enhancing oxidative phosphorylation (OXPHOS), and driving NET formation. In contrast, other studies suggest that CD177^+^ neutrophils facilitate transendothelial migration through the CD177–PR3–PECAM-1 molecular axis, preferentially infiltrating inflamed mucosal tissues and thereby promoting rapid barrier repair and inflammation attenuation [12].

**Figure 2:**
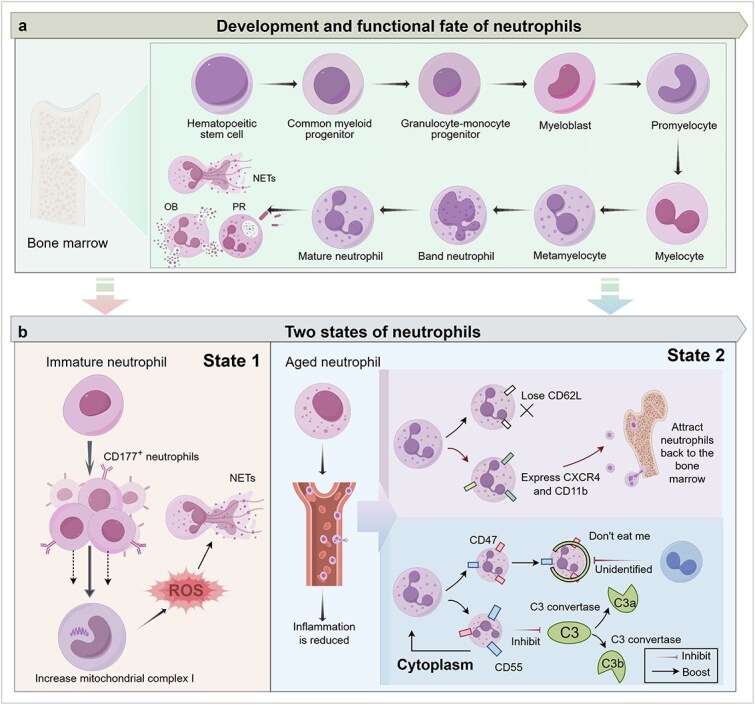
Developmental trajectory and functional states of neutrophils in ischaemia–reperfusion injury. (**a**) Development and functional fate of neutrophils. Neutrophils originate from haematopoietic stem cells (HSCs) and undergo stepwise differentiation through common myeloid progenitors (CMPs) and granulocyte–monocyte progenitors (GMPs), sequentially progressing through the myeloblast, promyelocyte, myelocyte, metamyelocyte, and band neutrophil stages before maturing into segmented neutrophils within the bone marrow. Fully mature neutrophils are equipped with effector functions, including the capacity to release NETs, which contribute to host defence and inflammatory responses. (**b**) Two functional states of neutrophils. Neutrophils exhibit dynamic state transitions. State 1 (immature/newly released neutrophils) is characterized by CD62L expression and low CXCR4 levels, facilitating their mobilization into the peripheral circulation. State 2 (aged neutrophils) is marked by loss of CD62L, upregulation of CXCR4 and CD11b, and expression of the ‘don’t eat me’ signal CD47, which together promote chemokine-guided homing back to the bone marrow. This state is associated with the presence of CD177^+^ subsets and enhanced NET formation. Aged neutrophils can exert anti-inflammatory effects by attenuating complement activation, including suppression of C3 convertase activity and reduced generation of C3a and C3b, partly through upregulation of CD55. Concurrently, increased mitochondrial complex I activity supports oxidative phosphorylation, providing the energy required for neutrophil trafficking and functional maintenance. *NETs* neutrophil extracellular traps, *IRI* ischaemia–reperfusion injury, *ROS* reactive oxygen species, *OXPHOS* oxidative phosphorylation, *PR3* proteinase 3, *PECAM-1* platelet endothelial cell adhesion molecule-1, *C3* complement component 3, *C3a* complement component 3a, *C3b* complement component 3b, *CXCR4* C-X-C motif chemokine receptor 4, *CD* cluster of differentiation

In summary, neutrophil behaviour during IRI is highly heterogeneous and shaped by developmental stage, polarization state, and dynamic interactions among distinct subsets, which together govern both tissue injury and repair (Figure 2).

### Mechanisms and context-dependent functions of NETs in IRI

NETs were first described by Brinkmann and colleagues in 2004 as filamentous, web-like structures composed of decondensed chromatin decorated with granular proteins. These components include myeloperoxidase (MPO), neutrophil elastase (NE), histones, and posttranslationally modified histones such as citrullinated histone H3 (CitH3) [13]. Functionally, NETs act as essential antimicrobial barriers under physiological conditions by capturing and neutralizing invading pathogens, including bacteria and fungi. However, in pathological contexts—such as sterile inflammation, thrombosis, and IRI—excessive or dysregulated NET formation can amplify tissue injury through potent pro-inflammatory and cytotoxic effects [13].

Mechanistically, current studies classify NET formation into two major pathways: lytic NETosis and non-lytic NETosis [14]. Lytic NETosis represents a distinct form of programmed cell death, mechanistically different from apoptosis and necrosis, and is driven by a defined molecular cascade involving NADPH oxidase (NOX)–dependent ROS accumulation, peptidyl arginine deiminase 4 (PAD4) activation, and histone citrullination [15]. These coordinated events promote chromatin decondensation and nuclear envelope breakdown, ultimately leading to plasma membrane rupture and the extracellular release of DNA–granule protein complexes. In sterile IRI, including renal and hepatic IRI, this pathway is predominantly initiated by endogenous DAMPs. In particular, during mild renal IRI, NOX-dependent signalling appears to be the principal driver of NET formation. The functional importance of this signalling axis is further supported by C3-deficient mouse models, in which the loss of C3 disrupts C3a–C3aR1 engagement, suppresses ERK/ROS/PAD4 signalling, and markedly attenuates renal tissue injury, neutrophil infiltration, and NETosis [16].

In contrast to the NOX-dependent lytic NETosis observed in renal and other settings, hepatic IRI follows a distinct regulatory paradigm in which upstream signalling is predominantly governed by platelet-derived sphingosine-1-phosphate (S1P). Through the differential engagement of S1PR2 and S1PR3, S1P exerts bidirectional control over non-lytic NETosis, with S1PR2 promoting and S1PR3 restraining NET formation [17]. This regulatory pathway operates independently of NOX activity but involves the modulation of autophagic flux and preferentially induces the rapid extrusion of mitochondrial DNA rather than nuclear DNA. Such an antagonistic signalling system has been identified in liver transplantation–associated IRI models as a critical determinant of susceptibility to NETosis.

Importantly, NET formation in IRI-associated conditions is not uniformly detrimental. In renal IRI, early and low-level NET production may facilitate activation of Transforming Growth Factor-beta (TGF-β) signalling, thereby promoting tubular epithelial cell proliferation and tissue repair and conferring a protective effect [18]. Beyond tissue repair, maintaining a controlled degree of NETosis that preserves neutrophil antimicrobial function can also be beneficial for host defence, as within the circulatory environment, NETs primarily act to capture invading microorganisms and limit their dissemination [19].

**Figure 3:**
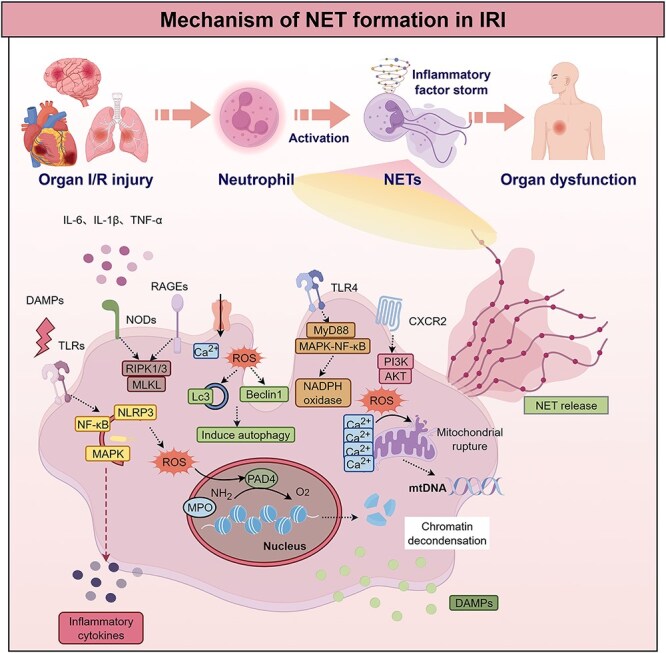
NET formation mechanisms in IRI. Organ ischaemia–reperfusion injury induces an inflammatory cytokine milieu that activates neutrophils and initiates NET formation. Damage-associated molecular patterns engage pattern-recognition receptors, including TLR4, RAGE, and NODs, thereby activating MyD88-dependent signalling and downstream PI3K–AKT, MAPK, NF-κB, and RIPK1/3–MLKL pathways. These signals promote NADPH oxidase activation, mitochondrial dysfunction, and excessive reactive oxygen species production. ROS accumulation, together with intracellular calcium flux, triggers NLRP3 inflammasome activation and autophagy, while PAD4-mediated histone citrullination drives chromatin decondensation. Granule proteins, including myeloperoxidase and neutrophil elastase, associate with nuclear and mitochondrial DNA, culminating in extracellular NET release. NETs subsequently amplify inflammation through the release of damage-associated signals and pro-inflammatory cytokines, ultimately contributing to organ dysfunction following ischaemia–reperfusion injury. *IRI* ischaemia–reperfusion, *NETs* neutrophil extracellular traps, *DAMPs* damage-associated molecular patterns, *TLR4* Toll-like receptor 4, *RAGE* receptor for advanced glycation end-products, *NODs* nucleotide-binding oligomerization domain receptors, *MyD88* myeloid differentiation primary response 88, *PI3K* phosphoinositide 3-kinase, *AKT* protein kinase B, *MAPK* mitogen-activated protein kinase, *NF-κB* nuclear factor kappa B, *RIPK1/3* receptor-interacting protein kinase 1/3, *MLKL* mixed lineage kinase domain-like protein, *ROS* reactive oxygen species, *NLRP3* NOD-like receptor family pyrin domain-containing 3, *PAD4* peptidyl arginine deiminase 4, *MPO* myeloperoxidase, *NE* neutrophil elastase, *mtDNA* mitochondrial DNA, *IL-6* interleukin-6, *IL-1β* interleukin-1 beta, *TNF-α* tumour necrosis factor-alpha

Despite these advances, how NETs generated in distinct anatomical contexts differ remains poorly defined. Variations in local microenvironmental cues that drive NET release, together with differences in the underlying mechanisms of NET formation, are likely to confer distinct cytotoxic properties [20]. Clarifying these context-dependent features is therefore essential for a more precise understanding of NET-mediated disease mechanisms and for the rational development of targeted therapeutic strategies [17] (Figure 3).

### Neutrophils and neutrophil extracellular traps in IRI

#### The endothelial–neutrophil axis in the initiation of IRI

Inflammation during IRI is initiated by tightly coordinated interactions between endothelial cells and neutrophils, which together constitute a central regulatory axis integrating conserved cross-organ mechanisms with tissue-specific molecular features [21]. During the early phase of reperfusion, vascular endothelial cells function as primary sensors of tissue injury. Abrupt oxygen reintroduction triggers the xanthine oxidase–mediated metabolism of hypoxanthine that accumulates during ischaemia, resulting in the generation of superoxide anions and the subsequent formation of hydroxyl radicals. These ROS activate endothelial NF-κB signalling, leading to the stepwise upregulation of the expression of adhesion molecules, including E-selectin, P-selectin, vascular cell adhesion molecule-1 (VCAM-1), and intercellular adhesion molecule-1 (ICAM-1), within hours. In parallel, DAMPs, such as high-mobility group box 1 (HMGB1) and histones released from necrotic cells, further amplify inflammatory signalling through endothelial Toll-like receptor 4/9 (TLR4/9)–myeloid differentiation primary response 88 (MyD88) signalling. The convergence of oxidative and innate immune signals promotes the endothelial secretion of chemokines, including IL-8 and C-X-C Motif Chemokine Ligand 1 (CXCL1), thereby establishing chemotactic gradients that guide neutrophil recruitment to sites of reperfusion injury [22].

Once the activated endothelium establishes a permissive inflammatory microenvironment, neutrophil recruitment occurs through a well-defined multistep cascade comprising rolling, firm adhesion, intravascular crawling, and transendothelial migration. The initial rolling phase is mediated by P-selectin and E-selectin, which facilitate the transient tethering of neutrophils to the vascular endothelium. This transient interaction is followed by stable adhesion and directional crawling, processes driven primarily by interactions between endothelial ICAM-1 and the neutrophil integrin Mac-1 (CD11b/CD18) [23]. Importantly, integrin activation during this transition is tightly regulated and depends on extracellular disulphide bond rearrangements. Endoplasmic reticulum protein 72 (Erp72) serves as a key regulator of integrin conformational dynamics, and Erp72 deficiency markedly impairs neutrophil adhesion and migration [24]. Collectively, these findings position neutrophil recruitment within a multilayered regulatory network that integrates redox control, conformational regulation, and functional execution, underscoring potential opportunities for precision intervention in oxidative stress–related disorders, including IRI [22].

Although this canonical recruitment paradigm applies broadly across vascular beds, hepatic IRI is associated with pronounced organ-specific deviations. The narrow diameter and low shear stress within hepatic sinusoids favour early neutrophil retention predominantly through mechanical trapping rather than selectin-mediated rolling [25]. Consistent with this haemodynamic context, sinusoidal endothelial cells lack selectin expression, and neutrophil adhesion is instead dominated by integrin-dependent mechanisms. This distinctive recruitment pattern provides a mechanistic explanation for the limited efficacy of anti-selectin therapies observed in clinical trials of hepatic IRI [25]. In addition to leukocyte recruitment, endothelial activation in this setting directly promotes NET formation. Injured sinusoidal endothelial cells release interleukin-33 (IL-33), which induces NET generation through the engagement of the ST2 receptor. In turn, NET-derived histones and DNA exacerbate endothelial injury by degrading the protective glycocalyx and exposing basement membrane adhesion sites, thereby establishing a self-amplifying positive feedback loop that links endothelial activation, NET release, and progressive endothelial damage [26].

**Figure 4:**
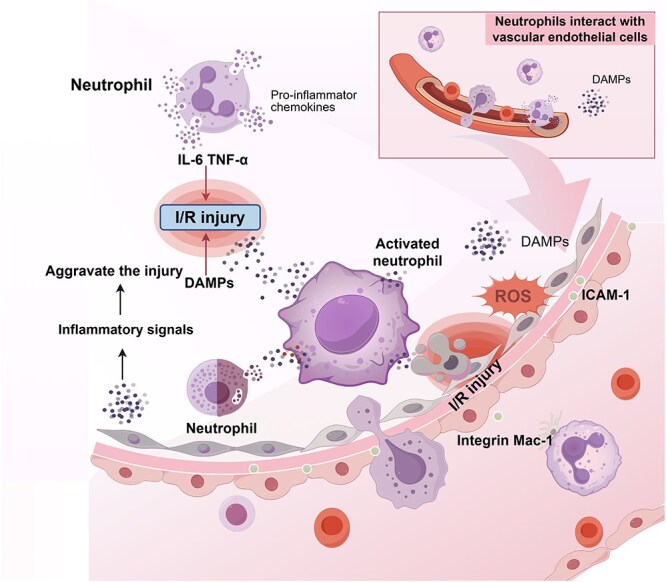
Neutrophil-endothelial interactions during IRI. IRI triggers the release of pro-inflammatory cytokines, such as IL-6 and TNF-α, along with DAMPs, which collectively activate neutrophils and facilitate their recruitment to the vascular endothelium. Upon arrival, neutrophils adhere to endothelial cells via Mac-1 integrin, which specifically binds ICAM-1 expressed on the endothelial surface. This binding enables firm adhesion and promotes directional crawling of neutrophils along the vascular wall. Once activated, neutrophils release ROS and further DAMPs, amplifying the inflammatory response and contributing to endothelial injury. This sustained inflammatory activity establishes a positive feedback loop that intensifies vascular damage, promotes endothelial dysfunction, and drives immune cell infiltration during IRI. *DAMPs* damage-associated molecular patterns, *ROS* reactive oxygen species, *ICAM-1* intercellular adhesion molecule-1, *IL-6* interleukin-6, *TNF-α* tumour necrosis factor-alpha, *IRI* ischaemia–reperfusion injury, *Mac-1* integrin macrophage-1 antigen

In addition to adhesion- and cytokine-driven mechanisms, postinfarction endothelial cells (ECs) actively shape early neutrophil recruitment through extracellular vesicle–mediated signalling. Following infarction, ECs exhibit enhanced extracellular vesicle (EC-EV) release, which contributes to the rapid accumulation of neutrophils within injured regions [27]. This noncanonical recruitment pathway operates independently of classical chemokine signalling and may account for the early, phase-specific neutrophil infiltration observed during IRI. Mechanistically, EC-EVs are enriched in miRNA-126-3p/5p and promote neutrophil transendothelial migration by facilitating interactions between VCAM-1 and neutrophil surface integrins [28]. Notably, EC-EVs display functional duality, simultaneously driving pro-inflammatory neutrophil recruitment while suppressing ferroptosis and preserving endothelial integrity. This apparent heterogeneity is likely attributable to distinct EV subpopulations with different cargo compositions. Nevertheless, current evidence remains limited by the absence of single-vesicle-resolution cargo profiling and conditional genetic models, which are needed to quantitatively define the contribution of this pathway to overall neutrophil recruitment during IRI [27] (Figure 4).

#### Neutrophil- and NET-mediated injury across parenchymal organs

After transendothelial migration, neutrophils directly interact with parenchymal cells—including cardiomyocytes, renal tubular epithelial cells, and hepatocytes—and thereby actively participate in tissue injury during IRI. In support of this concept, multiphoton intravital microscopy has revealed the rapid accumulation and highly dynamic spatiotemporal behaviour of neutrophils across multiple organs during the early phases of IRI [29, 30]. These real-time observations provide direct evidence for organ-specific neutrophil functions and lay the foundation for determining how neutrophil effector programs, including NET formation, shape parenchymal injury responses [31].

##### Neutrophil polarization and NET-driven injury in cardiac IRI

In the heart, neutrophils represent the earliest and most rapidly expanding inflammatory cell population following myocardial reperfusion. Their abundance peaks at ~24 h after reperfusion and subsequently returns to baseline levels by Day 3 [32, 33]. During this acute phase, pro-inflammatory N1-polarized neutrophils predominate and exacerbate myocardial injury through the release of ROS, IL-1β, IL-6, tumour necrosis factor-alpha (TNF-α), and NETs, thereby promoting cardiomyocyte apoptosis and amplifying local tissue damage [34].

Importantly, accumulating evidence indicates that N1 polarization is not solely a neutrophil-intrinsic process but is actively shaped by signals derived from injured cardiomyocytes [35]. In the context of cardiac IRI, ischaemic cardiomyocytes release miR-9-5p-enriched extracellular vesicles, which suppress Krüppel-like factor 4 (KLF4) expression in neutrophils and drive neutrophil polarization towards a pro-inflammatory phenotype. This bidirectional crosstalk further amplifies the production of ROS, IL-1β, IL-6, and NETs, establishing a feed-forward loop that accelerates cardiomyocyte apoptosis and aggravates myocardial injury [35].

Beyond cardiomyocyte–neutrophil communication, the inflammatory cardiac microenvironment provides additional cues that reinforce NET formation. Under inflammatory conditions, diverse cytokines and stress-related stimuli converge to activate neutrophils and promote NETosis. Recent studies have demonstrated that, following cardiac IRI, infiltrating M1-polarized macrophages drive neutrophil NETosis through the AT-Rich Interaction Domain 3A (ARID3A)-dependent regulation of the Thrombospondin-1 (THBS1)/CD47 axis [36]. This macrophage–neutrophil signalling axis exacerbates myocardial inflammation and promotes fibrotic remodelling. Consistent with this mechanism, mice with myeloid-specific ARID3A deletion exhibit markedly reduced myocardial neutrophil infiltration and NET formation. Nevertheless, the absence of validation in human cohorts currently limits the translational interpretation of these findings in the context of human cardiac IRI [36].

In addition to intercellular inflammatory signalling, cell-intrinsic metabolic perturbations critically shape neutrophil effector responses in cardiac IRI. The disruption of mitochondrial homeostasis has emerged as a key driver of widespread NET activation. RNA sequencing analyses have revealed a pronounced upregulation of Mitochondrial Calcium Uptake 3 (MICU3) expression in peripheral neutrophils from rats subjected to myocardial ischaemia–reperfusion injury (MIRI) [37]. MICU3, a critical component of the mitochondrial calcium uniporter complex, aberrantly interacts with the mitochondrial channel protein Voltage-Dependent Anion Channel 1 (VDAC1), thereby promoting the excessive formation of mitochondria-associated membranes [38]. This maladaptive interaction results in mitochondrial calcium overload, leading to mitochondrial dysfunction and the loss of homeostatic control. Notably, these alterations have also been validated in neutrophils isolated from patients with MIRI [39]. Collectively, these findings suggest that mitochondrial calcium dysregulation is a central mechanism linking neutrophil activation to NET formation in cardiac IRI and highlight mitochondrial signalling as a promising target for precision therapeutic intervention [40].

##### DAMP–NET amplification loops in renal IRI

In animal models of renal IRI, the initiation of NET formation is largely driven by DAMPs released from hypoxic and necrotic renal tubular epithelial cells. Under ischaemic conditions, tubular epithelial cells undergo programmed necrosis mediated by receptor-interacting protein kinase–dependent pathways [41]. As a consequence of cell death, necrotic tubules release DAMPs, including HMGB1 and histones, which bind to receptors on the neutrophil surface and act as key initiators of NET-associated inflammatory cascades. Through the activation of downstream intracellular signalling pathways, these DAMPs promote NETosis, thereby amplifying renal inflammation and aggravating tissue injury [42].

Beyond serving as downstream effectors of tubular injury, NETs themselves further propagate inflammatory damage. Following NET release, key NET-associated components—including MPO, NE, and histones—function rapidly as ‘mobile reservoirs’ of DAMPs [20]. Acting through convergent pathways that encompass mitochondrial dysfunction, proteolytic tissue injury, and immune activation, these mediators accelerate necroptotic cell death and initiate secondary inflammatory amplification within a self-reinforcing inflammatory loop. Consequently, endogenous DAMPs together with NET-derived effectors have emerged as central pathological drivers of renal inflammation in the context of IRI [43].

In parallel with DAMPs and NET-driven amplification loops, complement activation provides an additional layer of inflammatory reinforcement. Complement activation further amplifies neutrophil-driven inflammatory responses by reinforcing innate immune signalling. In this context, the complement fragments complement component 3a (C3a) and C5a bind to neutrophil receptors (CR1, CR3, and CR4), thereby potentiating downstream inflammatory cascades [44, 45]. Beyond this canonical pathway, recent studies have identified a distinct neutrophil subset with an N(IL-23^+^IL-18^+^) phenotype that expresses IL-17, major histocompatibility complex class II molecules, and costimulatory factors, enabling enhanced T-cell activation and promoting renal injury aggravation [46]. Although this subset has been characterized predominantly in transplantation models, its transient emergence during sterile IRI is biologically plausible given that injured renal epithelial cells can release IL-23 and IL-18. Future studies integrating spatial transcriptomics with intravital imaging will be essential for validating this hypothesis and delineating its pathophysiological relevance [46].

##### Neutrophil- and NET-mediated immunovascular injury in the liver

The liver is a distinctive immunological organ that simultaneously maintains immune tolerance and active immune surveillance, rendering it a critical site for the pathological actions of NETs [25]. Under stress conditions such as liver transplantation or hepatic resection, a broad spectrum of injury-associated signalling events involving hepatocytes, hepatic stellate cells, liver sinusoidal endothelial cells, and Kupffer cells is initiated [47]. Signals derived from these interacting cell populations converge to promote neutrophil activation and NET formation, thereby setting the stage for the initiation and progression of hepatic IRI [48].

Building on this immunological context, experimental models of hepatic IRI have demonstrated that neutrophil-driven inflammation is sustained by increased ICAM-1 expression and by the release of CXC chemokines (CXCL1/CXCL2) from liver sinusoidal endothelial cells and activated Kupffer cells. Although neutrophil activation and accumulation within hepatic sinusoids do not directly injure the epithelium, the subsequent release of NETs fundamentally alters the pathogenic role of neutrophils [48]. Core NET components, including CitH3 and NE, disrupt hepatocyte membrane integrity and induce mitochondrial oxidative stress and ferroptosis [49]. In parallel, NE impairs sinusoidal microcirculation by suppressing prostacyclin (PGI_2_) synthesis, thereby contributing to the hepatic ‘no-reflow’ phenomenon. Consistent with these mechanistic observations, longitudinal analyses of serum from liver transplant recipients have revealed a significant inverse correlation between postoperative NET levels and liver function indices, with peak NET concentrations independently predicting early graft dysfunction [50].

In addition to direct tissue injury, NETs also act as potent immunomodulatory signals within the hepatic immune microenvironment. Specifically, NETs promote Kupffer cell polarization towards a pro-inflammatory M1 phenotype by increasing HMGB1 release and activating the TLR4/MAPK signalling pathway, thereby establishing a feed-forward circuit that further amplifies NET formation. Importantly, this self-reinforcing inflammatory loop provides a mechanistic basis for therapeutic interventions [51, 52]. Accumulating evidence indicates that the pharmacological inhibition of key NET generation pathways significantly reduces serum alanine aminotransferase (ALT) and aspartate aminotransferase (AST) levels while attenuating hepatocyte apoptosis. Complementarily, the enzymatic degradation of the NET DNA scaffold using DNase I markedly decreases intrahepatic NET accumulation and NLRP3 inflammasome activation, effectively dampening the IL-1β/IL-18–driven cytokine storm [53, 54].

Taken together, these findings indicate that NETs should not be regarded merely as antimicrobial scaffolds but rather as central regulatory hubs coordinating inflammation, immune dysregulation, and microvascular injury throughout the course of liver transplantation [51]. In line with this expanded pathogenic role, the dynamic monitoring of circulating CitH3–DNA or MPO–DNA complexes has emerged as a promising integrated biomarker for predicting hepatic IRI. Beyond risk stratification, a staged and multitarget therapeutic strategy directed against NETs—encompassing the upstream blockade of danger-sensing pathways (TLR4/NF-κB), the midstream inhibition of PAD4 activity and ROS generation, and downstream enzymatic degradation using DNase I—has demonstrated synergistic efficacy in multiple preclinical models [55, 56]. These interventions collectively reduce ALT/AST levels, attenuate rejection responses, preserve microvascular integrity, and prolong graft survival. Looking ahead, the rational integration of NET-targeted approaches with established immunosuppressive regimens, together with the precise delineation of therapeutic timing and safety windows, may represent a pivotal step towards advancing liver transplantation from a technically successful procedure to a strategy capable of achieving durable, rejection-free graft tolerance [57].

##### NET-driven neurovascular dysfunction in cerebral IRI

The pathological progression of cerebral IRI follows a characteristic ‘double-hit’ paradigm in which neutrophils and NETs act as central drivers of a self-perpetuating inflammatory cycle. Ischaemic insult rapidly mobilizes neutrophils from the bone marrow and spleen, leading to excessive activation and the release of proteolytic enzymes and NETs [58]. This heightened neutrophil response promotes the recruitment and activation of additional peripheral immune cells as well as resident immune populations within the brain, thereby amplifying neuroinflammatory cascades. Notably, NET accumulation peaks ~3–5 days after stroke onset and directly impairs vascular remodelling through the activation of the cGAS–STING–type I interferon signalling pathway, resulting in reduced neovascularization and the exacerbated disruption of the blood–brain barrier [59]. Moreover, the DNA scaffold of NETs persistently stimulates endothelial immune responses through multiple mechanisms, establishing a self-reinforcing inflammatory loop that sustains and aggravates cerebral injury.

Beyond their structural and vascular effects, neutrophils further amplify postischemic neuroinflammation through the release of myeloid-related protein 14 (MRP14) [60]. By engaging TLR4 on microglia, MRP14 functionally reprograms microglial responses by simultaneously delivering ‘do not eat me’ and cytotoxic activation signals. This dual effect suppresses microglial phagocytic clearance, leading to prolonged neutrophil persistence and the concomitant activation of the NLRP3 inflammasome to induce pyroptosis and subsequent interleukin-1β (IL-1β) release [60]. IL-1β, in turn, feeds back on neutrophils, promoting the further mobilization of bone marrow–derived neutrophils and upregulation of MRP14 expression, thereby reinforcing a self-sustaining inflammatory circuit. Importantly, PAD4, an enzyme indispensable for NET formation, also regulates MRP14-driven inflammatory amplification, positioning PAD4 as a critical molecular hub linked to these convergent pathogenic pathways [60].

At the upstream mechanistic level, DAMPs released from injured brain tissue—most prominently HMGB1—serve as potent initiators of NET formation [61]. This pro-NETotic effect is mediated, at least in part, through interactions between the receptor for advanced glycation end-products (RAGE) and the β2 integrin Mac-1 (CD11b/CD18) on neutrophils [62]. Consistent with this initiating role, clinical studies have demonstrated that elevated circulating HMGB1 levels are strongly associated with poor functional outcomes in patients with ischaemic stroke. Experimental evidence further indicates that direct binding between RAGE and CD11b is sufficient to trigger NET release, although the downstream signalling events remain incompletely defined [63].

Collectively, the synergistic injury arising from these interconnected pathological dimensions provides a coherent mechanistic explanation for the clinical phenomenon termed ‘recanalization–outcome mismatch’. Even when vascular patency is successfully restored, the NET-mediated impairment of vascular repair and MRP14-driven neuroinflammation act in concert to reinforce one another, leading to the sustained deterioration of the neurovascular unit microenvironment [64]. Within this integrated pathogenic framework, NET induction via the DAMP–RAGE/Mac-1 axis constitutes the initiating trigger, whereas PAD4 functions as a central regulatory hub, thereby emerging as a promising therapeutic target for disrupting this self-perpetuating disease cascade [65].

##### NET-induced structural and inflammatory damage in pulmonary IRI

The excessive accumulation of NETs and their associated components has pronounced deleterious effects on lung tissue during IRI [66]. At the molecular and cellular levels, NET-derived histones display direct cytotoxicity towards endothelial cells, whereas MPO-generated ROS create a pro-injurious microenvironment that promotes epithelial apoptosis and necrosis [67]. Importantly, evidence from lipopolysaccharide-induced acute lung injury models indicates that the enzymatic degradation of the NET DNA scaffold by DNase I markedly enhances the alveolar macrophage-mediated phagocytosis of NET remnants, accompanied by a significant reduction in pro-inflammatory cytokines such as IL-6 and TNF-α. Notably, macrophage-driven NET clearance occurs without eliciting secondary pro-inflammatory cytokine release, supporting the therapeutic feasibility of strategies aimed at facilitating NET degradation and removal in inflammatory lung injury [67].

Accumulating evidence has indicated that NETs also have profound effects on lung structure and function. Through their cytotoxic components, NETs directly injure alveolar epithelial cells and induce necroptotic cell death, a process increasingly recognized as a key driver of acute lung injury progression [68]. Beyond direct cellular toxicity, NETs disrupt pulmonary tissue architecture by promoting alveolar fusion and collapse, thickening of the alveolar septa, and extensive inflammatory cell infiltration, thereby exacerbating lung pathology. Consistent with these mechanistic insights, *in vivo* studies have demonstrated a strong association between elevated NET levels and the progressive deterioration of lung function in lung transplant recipients, leading to a vicious cycle characterized by heightened susceptibility to infection and impaired tissue repair [69]. Collectively, these findings further support the rationale for therapeutic strategies that inhibit NET formation or enhance NET clearance—particularly through the augmentation of macrophage-mediated NET removal—as potential interventions for both acute lung injury and lung transplantation–related complications [70].

##### Shared NET-driven pathogenic mechanisms across other organs

In inflammatory bowel disease, components of NETs, including histones and MPO, directly injure intestinal epithelial cells [71]. Beyond this direct cytotoxicity, double-stranded DNA released from NETs activates the TLR9/cGAS–STING signalling axis, thereby sustaining the production of pro-inflammatory cytokines such as IL-1β and TNF-α and establishing a self-perpetuating cycle of ‘NETs–inflammation–tissue injury’. Consistent with this pathogenic loop, NET levels are markedly elevated in both intestinal tissues and the circulation of patients with inflammatory bowel disease and correlate positively with disease activity [72]. In parallel, excessive NET accumulation contributes to an increased risk of thromboembolic complications in this population. A mechanistically analogous process has been described in severe COVID-19, where SARS-CoV-2 directly and indirectly promotes NET formation, leading to immunothrombus development, microvascular occlusion, and subsequent organ failure. Notably, NET-driven immunothrombosis represents a double-edged response, as it not only supports haemostasis and limits bleeding but also contributes to pathogen containment and immune defence during later stages of disease [72].

At the level of NET-associated effector molecules, matrix metalloproteinase-9 (MMP9) has emerged as a key granular protein released during NET formation and is an important mediator of IRI. Following NET release, the expression of MMP9 can further increase through activation of the extracellular signal-regulated kinase (ERK) signalling pathway, thereby establishing a self-amplifying NET–MMP9 feedback loop [73]. Despite this mechanistic insight, whether upregulated MMP9 expression acts as an initiating driver of NET formation or arises predominantly as a downstream consequence of NET generation remains unresolved, highlighting an important gap in the current understanding of NET-associated protease signalling [74].

In addition to protease-mediated injury, ROS and lipid peroxidation constitute additional, tightly interconnected mechanisms through which NETs induce endothelial cell death and microvascular dysfunction. In intestinal endothelial cells, NETs promote the phosphorylation of FUN14 domain-containing protein 1 (FUNDC1) at Tyr18, thereby suppressing mitophagy and disrupting mitochondrial quality control. In addition to the vasculature, ROS play a central role in tissue injury during skeletal muscle IRI [75]. In this context, the principal sources of ROS include mitochondria, NOX, and xanthine oxidase. Excessive ROS accumulation damages mitochondrial membranes and respiratory chain complexes, triggers the opening of the mitochondrial permeability transition pore, induces mitochondrial swelling, and ultimately culminates in apoptotic cell death [76].

**Figure 5:**
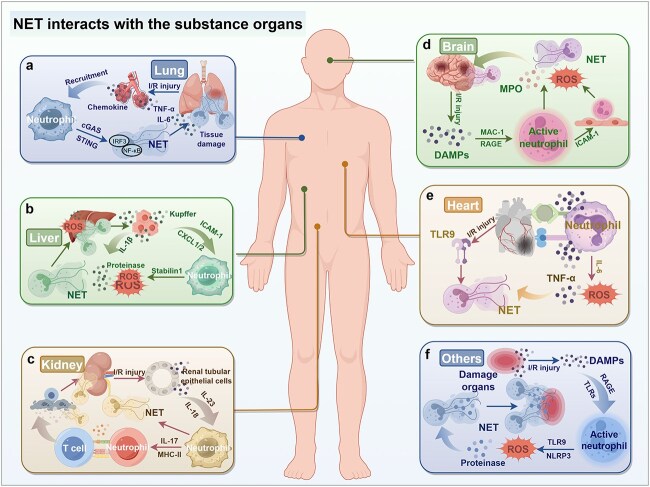
NET-mediated interactions with parenchymal organs during IRI. (**a**) Lung: During IRI, NETs promote the secretion of pro-inflammatory cytokines and chemokines (such as TNF-α and IL-6) through the cGAS–STING pathway, as well as the nuclear translocation of IRF3 and NF-κB, thereby mediating lung ischaemia–reperfusion injury. (**b**) Liver: In the liver, NETs interact with Kupffer cells via ICAM-1 and chemokines such as CXCL1/CXCL2, promoting neutrophil recruitment. NET-derived proteases and ROS cause hepatocellular damage. Molecules such as Stabilin-1 and IL-17 further modulate the inflammatory microenvironment. (**c**) Kidney: Renal tubular epithelial cells respond to IRI by interacting with NETs, triggering the expression of IL-17 and MHC class II molecules. These responses facilitate T-cell activation and contribute to sustained renal inflammation and tubular injury. (**d**) Brain: NETs promote neuroinflammation by interacting with Mac-1 and RAGE receptors on neural and immune cells. DAMP-mediated neutrophil activation results in excessive ROS production and NET release, further amplifying neuronal damage. (**e**) Heart: In the myocardium, NETs activate neutrophils via the TLR9 signalling pathway, leading to the release of TNF-α and ROS. These inflammatory mediators exacerbate myocardial damage and dysfunction following reperfusion. (**f**) Other organs: In various organs affected by IRI, recruited neutrophils lead to excessive production of ROS and release of NETs through activation of TLR9 and NLRP3, ultimately resulting in tissue damage. *NETs* neutrophil extracellular traps, *IRI* ischaemia–reperfusion injury; *MPO* myeloperoxidase, *NLRP3* nucleotide-binding domain, leucine-rich repeat containing family, pyrin domain-containing 3, *IL-17* interleukin-17, *TLR9* toll-like receptor 9, *DAMPs* damage-associated molecular patterns, *ROS* reactive oxygen species, *TNF-α* tumour necrosis factor-alpha, *NF-κB* nuclear factor kappa-light-chain-enhancer of activated B cells, *PI3K* phosphatidylinositositol-4,5-bisphosphate 3-kinase, *AKT* protein kinase B, *CXCL1/CXCL2* C-X-C motif chemokine ligand 1/2, *RAGE* receptor for advanced glycation end-products, *ICAM-1* intercellular adhesion molecule-1, *IRF3* interferon regulatory factor 3

Collectively, these observations underscore the multifaceted roles of neutrophils and NETs as shared pathological amplifiers across diverse organ systems in ischaemia–reperfusion and inflammatory injury [77]. Despite considerable progress, substantial gaps remain in our understanding of the regulatory hierarchies governing NET formation and the dynamic interplay between immune signalling and cellular metabolism [51]. In this context, continued advances in spatial multiomics, lineage tracing, and *in vivo* imaging technologies are expected to provide critical mechanistic insights and facilitate the development of increasingly precise, organ-specific therapeutic strategies in the coming years (Fig. 5).

### NETs as mediators of remote organ injury after IRI

Remote organ injury represents a pivotal step in the progression from IRI to multiple organ dysfunction syndrome [78]. In this context, recent advances have led to the concept of a ‘NET–organ axis’, which positions NETs as a mechanistic bridge linking localized inflammatory responses to dysfunction in distant organs. Following IRI, the excessive release of DAMPs activates the TLR4/MyD88 signalling pathway, thereby promoting NET formation. Consistent with this upstream mechanism, Zhuang and colleagues reported a positive correlation between circulating NET levels and the severity of renal IRI, implicating NETs as key mediators of remote organ injury [79]. In addition to amplifying local inflammation, NETs also interact with platelets and endothelial cells to promote immunothrombosis. This pathogenic pathway was further substantiated by Nakazawa *et al.* [80], who demonstrated that NET-driven thrombosis constitutes a critical mechanistic link between acute kidney injury (AKI) and secondary lung injury. Notably, the pharmacological inhibition of NET formation significantly attenuates secondary organ damage, underscoring the therapeutic relevance of targeting NETs in IRI-associated remote organ injury.

Extending these observations from systemic injury to organ-specific settings, evidence from lung transplantation–associated ischaemia–reperfusion injury models further substantiates the pathogenic contribution of NETs to remote organ injury [81]. In these models, the upregulation of the expression of NET-related genes is closely correlated with graft dysfunction, strengthening the association between sustained NET activity and impaired organ performance [82]. Beyond transcriptional signatures, components released from NETs—particularly histones—exert direct cytotoxic effects on alveolar epithelial cells and renal tubular cells, thereby aggravating tissue injury at distant sites. Importantly, preclinical studies have demonstrated that therapeutic strategies targeting NETs, including PAD4 inhibition and DNase I–mediated NET degradation, can effectively attenuate multiorgan injury and improve outcomes, underscoring their translational potential [67].

Collectively, these findings establish the ‘NET–organ axis’ as a unifying conceptual framework for elucidating the mechanisms underlying remote organ injury during IRI. Through the induction of direct structural cytotoxicity and the amplification of maladaptive immune responses, NETs have emerged as key drivers of multiple organ dysfunction syndrome [81]. Consequently, therapeutic strategies aimed at modulating NET formation or activity hold substantial promise for the prevention and management of remote organ injury in the context of IRI.

### Neutrophil-targeted therapeutic strategies in IRI

Accumulating evidence indicates that neutrophils and NETs are central effectors of tissue injury during IRI, indicating that neutrophil-targeted interventions are attractive therapeutic options [83, 84] (Table 1). Accordingly, current research has increasingly focused on strategies that modulate neutrophil activation and restrain NET formation to mitigate IRI severity. Preclinical investigations have explored a broad therapeutic spectrum, encompassing C5a receptor antagonists, mitochondria-targeted antioxidants, PAD4 inhibitors, inhibitors of key intracellular signalling pathways, and anti-inflammatory and anticoagulant therapies [85–90]. In conjunction with approaches that interfere with neutrophil activation or NET-associated signalling cascades, exogenous DNase administration has emerged as a rational strategy to accelerate NET degradation [58].

**Figure 6:**
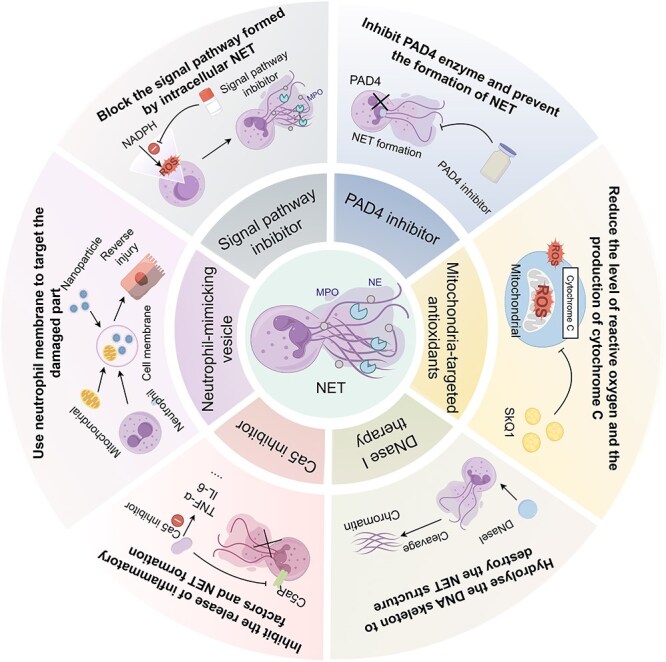
Therapeutic strategies targeting NETs and their pathological effects. A range of strategies have emerged to inhibit NET formation or alleviate its pathological consequences. PAD4 inhibitors suppress histone citrullination, blocking chromatin decondensation and NET release. Signal pathway inhibitors, such as those targeting NADPH oxidase–mediated ROS production, reduce NET formation and downstream inflammatory signalling (e.g. NF-κB, Nrf2). Neutrophil-mimicking vesicles deliver targeted therapies to inflamed tissues. C5a receptor antagonists bind to C5aR, inhibiting downstream inflammatory signalling and reducing NET formation and inflammatory cytokine release. DNase I therapy degrades extracellular DNA, dismantling NET structures and mitigating inflammation. Mitochondria-targeted antioxidants (e.g. SkQ1) lower mitochondrial ROS and cytochrome *c* release, attenuating NET-driven oxidative stress. These multifaceted approaches offer therapeutic potential for NET-associated diseases such as ischaemia–reperfusion injury, autoimmunity, and thrombosis. *NETs* neutrophil extracellular traps, *IRI* ischaemia–reperfusion injury, *ROS* reactive oxygen species, *TNF-α* tumour necrosis factor-alpha, *PAD4* peptidylarginine deiminase type 4, *NF-κB* nuclear factor kappa-light-chain-enhancer of activated B cells, *Nrf2* nuclear factor erythroid 2-related factor 2, *NADPH* nicotinamide adenine dinucleotide phosphate, *C5aR* complement component 5a receptor

However, the therapeutic manipulation of NETs is inherently complex. NETs play an indispensable role in antimicrobial host defence, and the excessive suppression of NET formation or indiscriminate NET degradation may result in unintended immunological consequences [91]. Moreover, DNA and histones—the principal structural components of NETs—also function as DAMPs and, if inadequately cleared, can further amplify inflammatory signalling [92]. Collectively, these considerations underscore the need for neutrophil-targeted strategies that carefully balance the preservation of essential immune functions with the limitation of inflammation-driven collateral tissue damage (Figure 6).

**Table 1:** Research progress on NET inhibitors in ischemia–reperfusion injury

Treatment	Mechanism	Disease	Therapeutic efficacy	Classification	Refs
DNase I nanoparticles	The principle of enzyme–substrate chemotaxis targets NETs while simultaneously clearing ROS and protecting mitochondria	AIS	Alleviating reperfusion injury, reducing infarct volume, and remodelling the microenvironment of the neurovascular unit	DNase I therapy	[58]
GSK484 nanoparticles	The drug GSK484 is encapsulated in nanoparticles by conjugating a neutrophil-selective binding peptide with a ROS-responsive polymer	TBI	Reducing the severity of brain injury and cerebral oedema, decreasing the infarct area, and reducing the extent of blood–brain barrier damage	PAD4 inhibitor	[86]
Targeted inhibition of mitochondrial complex I in CD177^+^ neutrophils	Selective blockade of mitochondrial respiratory chain complex I in CD177^+^ neutrophils, reducing their ROS production and NET formation	PIRI	Alleviating pulmonary oedema	Neutrophil membrane–based biomimetic vesicle therapy	[70]
Leukotriene C4 inhibition	Inhibition of leukotriene C4 synthesis or action reduces the activation and migration of neutrophils	MIRI	Reducing the infarct area	Cell signalling pathway inhibitor	[87]
Inhibition of lactate-induced mitochondrial calcium uptake	Lactate promotes the formation of NETs by increasing mitochondrial calcium uptake, thereby exacerbating damage	MIRI	Reduce the formation of NETs and alleviate MI/RI injury	Cell signalling pathway inhibitor	[40]
Polyprenol-loaded nanoparticles disguised as neutrophil extracellular vesicles	Nanoparticles disguised with NEVs enhance the targeting and penetration of drugs in the ischaemia–reperfusion injury area of the brain, reducing brain damage	CIRI	Improving neurological deficits after cerebral ischaemia–reperfusion, reducing infarct volume, and decreasing brain tissue inflammatory response	Neutrophil membrane-based biomimetic vesicle therapy	[88]
Inhibition of miR-9-5p activity or blockade of its induced neutrophil N1 polarization	miR-9-5p from cardiomyocyte-derived exosomes induces neutrophil N1 polarization, promotes the formation of NETs, and exacerbates tissue injury	MIRI	Inhibiting miR-9-5p or blocking its induced neutrophil N1 polarization can alleviate MIRI injury	Cell signalling pathway inhibitor	[35]
Human C5a receptor antagonist	Blocking C5a receptor to inhibit complement-mediated neutrophil activation and inflammation	HIRI	Protecting against hepatic ischaemia-reperfusion injury in rats	C5a receptor antagonis	[93]
Intervention targeting MMP9^^^high neutrophils	Reducing the MMP9^^^high neutrophil subset and the formation of NETs by modulating the expression of the transcription factor SPI1 and its downstream target gene CST7	MIRI	Improving cardiac function after MIRI and reducing myocardial injury.	Cell signalling pathway inhibitor	[89]
Inhibition of ERK (extracellular signal-regulated kinase)	Protecting SH-SY5Y cells from OGD/R injury by downregulating autophagy through alleviating mitochondrial fission	OGD/R	Reducing cellular damage, enhancing cell survival, and improving cell function	Cell signalling pathway inhibitor	[90]

*ROS* reactive oxygen species, *NETs* neutrophil extracellular traps, *AIS* acute ischaemic stroke, *PAD4* peptidylarginine deiminase 4, *NEVs* neutrophil extracellular vesicles, *MIRI* myocardial ischaemia–reperfusion injury, *CIRI* cerebral ischaemia–reperfusion injury, *OGD/R* oxygen–glucose deprivation/reperfusion injury, *PIRI* pulmonary ischaemia–reperfusion injury, *TB*I traumatic brain injury, *C5a* complement component 5a

#### C5a receptor antagonists

Among the upstream regulators of neutrophil activation, the complement system plays a pivotal role in the pathogenesis of IRI [93]. In particular, complement component C5a and its receptor (C5aR) form a critical node within the ‘complement–neutrophil–NET’ axis. C5a acts as a potent neutrophil activator and drives chemotaxis, degranulation, and NET formation, thereby serving as a key upstream trigger of NETosis [94]. On this mechanistic basis, the pharmacological blockade of C5aR has been shown to suppress pro-inflammatory cytokine release and may indirectly limit NET formation, enabling intervention at multiple levels of the inflammatory cascade during IRI [95]. Notably, representative C5aR antagonists have progressed into clinical trials. The selective small-molecule C5aR antagonist avacopan received FDA approval in 2021 for the treatment of Anti-Neutrophil Cytoplasmic Antibody-Associated Vasculitis (AAV) on the basis of landmark phase III ADVOCATE trial results [96]. In this randomized, double-blind study that enrolled 330 patients, avacopan demonstrated remission rates comparable to those of a prednisone tapering regimen at Week 26; however, it resulted in superior sustained remission rates at Week 52. Importantly, compared with standard glucocorticoid therapy, avacopan therapy was associated with better recovery of renal function and a significantly reduced risk of relapse [97]. These clinical findings provide compelling validation for targeting the C5a–neutrophil–NET axis in inflammatory diseases characterized by neutrophil-driven tissue injury, including IRI.

#### Mitochondria-targeted antioxidants

In addition to complement-driven neutrophil activation, intracellular metabolic stress signalling critically shapes NET formation during IRI. Mitochondrial reactive oxygen species (mtROS) are key initiators of NET formation, particularly in the context of vital NETosis. During IRI, mitochondrial dysfunction–induced oxidative stress accelerates NET generation and exacerbates tissue damage [98]. In this context, mitochondrion-targeted antioxidants, such as Plastoquinonyl-decyl-triphenylphosphonium Bromide (SkQ1), have been developed to selectively neutralize mtROS and have demonstrated robust organ-protective effects across multiple IRI models [99].

In addition to attenuating mitochondrial ROS accumulation and lipid peroxidation, SkQ1 limits cytochrome *c* release, thereby preserving cellular integrity and organ function. Nevertheless, the therapeutic efficacy of SkQ1 is highly dose dependent, as excessive concentrations may paradoxically exert pro-oxidant effects [100]. To increase therapeutic precision while minimizing adverse outcomes, future strategies should integrate smart controlled-release nanocarriers with real-time redox monitoring technologies. Such approaches may enable spatiotemporally controlled antioxidant delivery and more effective NET formation modulation [101].

#### PAD4 inhibitors

At the level of chromatin remodelling, histone citrullination represents a critical step in NET formation. PAD4, which serves as the core enzymatic regulator of this process, promotes chromatin decondensation and DNA release by catalysing the conversion of arginine residues to citrulline [102]. Irreversible PAD4 inhibitors, such as Cl-amidine, block NET release via covalent binding to the enzymatic active site, whereas the selective inhibitor GSK484 effectively suppresses NET formation, attenuates pulmonary oedema, and improves survival in *Streptococcus pneumoniae*–induced sepsis models [103].

In renal IRI, PAD4 inhibition ameliorates renal dysfunction by limiting histone citrullination and may reduce the risk of secondary cardiovascular complications. Despite these encouraging findings, the clinical translation of early PAD4 inhibitors, including GSK484 and Cl-amidine, remains limited by suboptimal pharmacokinetic properties and limited target specificity [104]. Specifically, compounds such as GSK484 exhibit low oral bioavailability and short half-lives, restricting their practical application in IRI treatment.

To overcome these obstacles, JBI-589 has been developed as a novel highly selective, reversible PAD4 inhibitor. Through an optimized molecular structure, this compound has been shown to significantly improve drug metabolism and pharmacokinetic characteristics, demonstrating excellent oral bioavailability and outstanding target specificity in mouse models [105]. These properties suggest that compared with early-generation inhibitors, JBI-589 has superior clinical translational prospects, providing a new candidate for PAD4-targeted therapy in IRI [106].

#### Intracellular signalling pathway inhibitors

Tofacitinib is a JAK–STAT signalling pathway inhibitor that blocks downstream signal transducer and activator of transcription (STAT) phosphorylation and activation through the selective inhibition of Janus kinase (JAK) [107]. In lupus-prone mice, tofacitinib significantly suppresses NET formation and ameliorates vascular dysfunction. A phase I double-blind, randomized safety trial further demonstrated that tofacitinib reduces circulating NET complex (MPO–DNA) levels and alleviates arterial stiffness in patients with systemic lupus erythematosus, suggesting the clinical translational potential of tofacitinib for its inhibitory effects on NETs [108].

Of greater significance is anifrolumab—a monoclonal antibody targeting type I interferon receptor subunit 1—which successfully achieved its primary endpoint in a phase III clinical trial, with a BILAG-based Composite Lupus Assessment response rate of 47.8% at Week 52 (vs. 31.5% with placebo, *P* = .001), and significantly improved skin lesions and enabled glucocorticoid reduction, becoming the first approved type I interferon pathway inhibitor and establishing a clinical pathway for indirectly modulating NETs by blocking upstream cytokine signalling [107, 109].

#### DNase I therapy

In contrast to strategies that inhibit NET formation, the direct degradation of extracellular DNA represents an alternative approach to limiting NET-mediated damage [98]. DNase I, a nonspecific endonuclease capable of degrading extracellular DNA, has been extensively investigated as a means to promote NET clearance. The clinical feasibility of this strategy has been validated in cystic fibrosis: recombinant human DNase I was approved in 1993 and, when administered via nebulization, degrades the abnormally accumulated NET DNA scaffold in the airways, significantly reducing sputum viscosity, improving pulmonary function, and decreasing acute exacerbations of respiratory infections. This successful case established DNase I as a pharmacological paradigm for NET-targeted therapy [110].

In the field of IRI, preclinical studies have demonstrated that exogenous DNase I administration can reduce myeloperoxidase and neutrophil elastase levels, improve tissue perfusion, and attenuate fibrosis in skeletal muscle IRI [111]. Recent studies have further expanded the applicability of this strategy: in an acute respiratory distress syndrome model, intravenous rhDNase significantly reduced platelet–NET aggregation, restored normal clotting time, and improved pulmonary microcirculatory perfusion by degrading the NET DNA scaffold, suggesting that the shift in administration strategy from nebulized inhalation to intravenous injection could substantially expand its therapeutic window, which holds important reference value for the treatment of multiorgan IRI [112].

#### Neutrophil membrane–based biomimetic vesicle therapy

On the basis of advances in targeted delivery, biomimetic nanovesicles derived from neutrophil membranes provide an active targeting strategy for IRI lesions. In this context, Ma *et al.* [113] demonstrated that encapsulating the anti-inflammatory cytokine IL-37 within neutrophil membrane–derived vesicles (N-MV@IL-37) not only enhances cytokine stability but also enables targeted delivery to the injured endothelium through P-Selectin Glycoprotein Ligand-1 (PSGL-1)-mediated recognition. In renal IRI models, this targeted approach has been shown to significantly reduce apoptosis and inflammatory responses, supporting its therapeutic efficacy [113].

Consistent with these findings, myocardial IRI models have further revealed that vesicles generated by the fusion of neutrophil membranes with functional mitochondria possess regenerative capacity and promote tissue repair. Despite these promising results, clinical translation will require the systematic evaluation of long-term biocompatibility and immunogenicity, particularly in chronic IRI settings. In parallel, the artificial intelligence–assisted optimization of membrane protein composition may further increase vesicle stability and targeting precision [113]. Collectively, these advances underscore the potential of neutrophil membrane–based biomimetic vesicles as personalized, multiorgan therapeutic platforms for IRI-associated pathologies.

#### Anti-inflammatory and immunomodulatory therapies

In addition to direct interference with NET formation or clearance, broader immunomodulatory strategies have been explored. NETs are indispensable for effective pathogen clearance; however, their protective function depends on a tightly regulated physiological balance. When NET formation becomes excessive or dysregulated, particularly under inflammatory conditions, it shifts from host defence to a major driver of secondary tissue injury. Accordingly, therapeutic strategies aimed at modulating neutrophil activation and NET production have garnered increasing interest [114].

Among these approaches, macrolide antibiotics such as azithromycin attenuate NET formation by suppressing the neutrophil respiratory burst. *In vitro* studies have demonstrated that pretreatment with azithromycin (50 μg/ml) markedly inhibits phorbol 12-myristate 13-acetate–induced ROS generation, providing a mechanistic basis for its immunomodulatory effects [115]. These experimental observations are reinforced by clinical evidence. In patients with bronchiectasis, long-term low-dose azithromycin therapy is associated with significantly reduced sputum NET levels accompanied by improvements in disease severity [116]. Similarly, early azithromycin administration in sepsis-associated AKI is correlated with concurrent reductions in NET burden and a lower incidence of renal complications [117]. Nevertheless, because NETs constitute an integral component of host immune defence, therapeutic inhibition must be carefully individualized according to disease context and severity to avoid the risks associated with excessive immunosuppression.

#### Anticoagulant therapy

The intimate interplay between NETs and coagulation pathways has prompted interest in anticoagulant-based interventions. Heparin is a widely used anticoagulant with well-established efficacy; however, its capacity to regulate NETs is limited by inherent molecular pleiotropy. At low doses, heparin suppresses NET formation, histone release, and the production of pro-inflammatory mediators [118]. In contrast, higher doses paradoxically increase NET generation and thrombosis through the formation of platelet factor 4 (PF4)–heparin complexes. This narrow therapeutic window, together with opposing dose-dependent effects, makes precise modulation particularly challenging in complex pathological settings characterized by multisystem dysregulation, such as sepsis [119].

By comparison, recombinant human soluble thrombomodulin (rhTM) inhibits histone-induced NET formation via its lectin-like domain independent of its anticoagulant activity, thereby circumventing the dose-related paradox observed with heparin [120].

*In vivo* studies have demonstrated that rhTM treatment reduces NET formation; in rats with septic shock, it also decreases congestion, inflammation, oedema, and bleeding.

**Figure 7:**
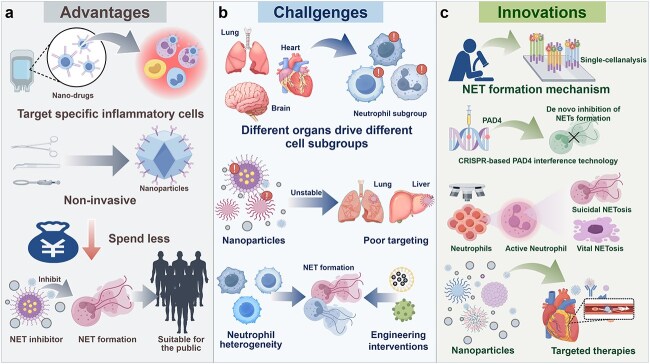
Key opportunities, barriers, and emerging approaches for targeting NETs. (**a**) Advantages: Nanomedicines enable selective targeting of inflammatory cells with noninvasive delivery, reducing costs and improving accessibility. Inhibition of NETs may prevent inflammation-related tissue injury. (**b**) Challenges: Organ-specific neutrophil subsets and cellular heterogeneity complicate targeting. Nanoparticle instability may cause off-target effects, particularly in the lungs and liver. Platform engineering remains complex. (**c**) Innovations: Single-cell tools are clarifying the mechanisms of NET formation. CRISPR/Cas9-mediated targeting of the PAD4 gene blocks NET formation at its source, offering a precise therapeutic strategy for ischaemia–reperfusion injury. Therapies targeting suicidal NETosis and engineered nanoparticles for state-specific modulation of NETs offer precision strategies. *NETs* neutrophil extracellular traps, *PAD4* peptidylarginine deiminase 4

Despite these encouraging findings, the clinical utility of rhTM remains a subject of debate; concerns include its high cost, the limitations imposed by intravenous administration, and the uncertain benefit–risk profile in patients without overt coagulopathy. Future studies should directly compare rhTM with dose-optimized low-molecular-weight heparin in NET-associated organ injury and explore biomarker-guided precision treatment strategies, such as those based on plasma histone levels [118] (Figure 7).

## Conclusions

The critical roles of neutrophils and NETs in IRI are now well established; however, translating these mechanistic insights into effective clinical treatments remains a significant challenge. A core limitation of the current research lies in the predominance of animal model studies, with relatively scarce human clinical data. The efficacy of most therapeutic strategies discussed in this review has been primarily demonstrated in rodent or large-animal models, whereas clinical validation in IRI patients remains limited. Furthermore, current research still lacks characterizations of neutrophil subsets and NET features in specific populations, particularly children and elderly individuals; however, the results of existing studies concerning neutrophil function, NET release patterns, and their associated functions following release remain unclear. Essentially, NETs formed under different pathological environments exhibit fundamental differences in initiating signalling and downstream biological consequences, yet the lack of systematic comparative analysis of these distinct processes constrains the development of targeted regulatory strategies. Several emerging strategies are beginning to expand therapeutic prospects, particularly CRISPR/Cas9-based systems, which offer transformative opportunities to intervene in NET formation through the selective disruption or modification of the PAD4 gene. However, the clinical translation of such strategies remains constrained by critical challenges, including efficient tissue-targeted delivery, the minimization of off-target genomic modifications, and the guarantee of long-term safety. Clearly, integrating neutrophil types and NET characteristics across different populations will provide clinical translation opportunities for precision medicine centred on NET-targeted regulation.

## References

[1] Hoste EAJ, Kellum JA, Selby NM, Zarbock A, Palevsky PM, Bagshaw SM, et al. Global epidemiology and outcomes of acute kidney injury. Nat Rev Nephrol. 2018;14:607–25. 10.1038/s41581-018-0052-0. 30135570

[2] Bonneau S, Landry C, Begin S, Adam D, Villeneuve L, Clavet-Lanthier MÉ, et al. Correlation between neutrophil extracellular traps (NETs) expression and primary graft dysfunction following human lung transplantation. Cells. 2022;11:3420. 10.3390/cells11213420. 36359815 PMC9656095

[3] Sayah DM, Mallavia B, Liu F, Ortiz-Muñoz G, Caudrillier A, DerHovanessian A, et al. Neutrophil extracellular traps are pathogenic in primary graft dysfunction after lung transplantation. Am J Respir Crit Care Med. 2015;191:455–63. 10.1164/rccm.201406-1086OC. 25485813 PMC4351593

[4] Gysemans C, Beya M, Pedace E, Mathieu C. Exploring neutrophil heterogeneity and plasticity in health and disease. Biomedicines. 2025;13:597. 10.3390/biomedicines13030597. 40149573 PMC11940349

[5] Shen X, Zhao Y, Wang Z, Shi Q. Recent advances in high-throughput single-cell transcriptomics and spatial transcriptomics. Lab Chip. 2022;22:4774–91. 10.1039/D2LC00633B. 36254761

[6] Khoyratty TE, Ai Z, Ballesteros I, Eames HL, Mathie S, Martín-Salamanca S, et al. Distinct transcription factor networks control neutrophil-driven inflammation. Nat Immunol. 2021;22:1093–106. 10.1038/s41590-021-00968-4. 34282331 PMC7611586

[7] Ng LG, Ostuni R, Hidalgo A. Heterogeneity of neutrophils. Nat Rev Immunol. 2019;19:255–65. 10.1038/s41577-019-0141-8. 30816340

[8] Hsu AY, Huang Q, Pi X, Fu J, Raghunathan K, Ghimire L, et al. Neutrophil-derived vesicles control complement activation to facilitate inflammation resolution. Cell. 2025;188:1623–41.e26. 10.1016/j.cell.2025.01.021. 39938514 PMC11934499

[9] Zhang D, Chen G, Manwani D, Mortha A, Xu C, Faith JJ, et al. Neutrophil ageing is regulated by the microbiome. Nature. 2015;525:528–32. 10.1038/nature15367. 26374999 PMC4712631

[10] Klempner MS, Gallin JI. Separation and functional characterization of human neutrophil subpopulations. Blood. 1978;51:659–69. 10.1182/blood.V51.4.659.659. 564724

[11] Deng H, Hu N, Wang C, Chen M, Zhao MH. Interaction between CD177 and platelet endothelial cell adhesion molecule-1 downregulates membrane-bound proteinase-3 (PR3) expression on neutrophils and attenuates neutrophil activation induced by PR3-ANCA. Arthritis Res Ther. 2018;20:213. 10.1186/s13075-018-1710-0. 30236159 PMC6148996

[12] Zheng C, Li J, Chen H, Ma X, Si T, Zhu W. Dual role of CD177 + neutrophils in inflammatory bowel disease: a review. J Transl Med. 2024;22:813. 10.1186/s12967-024-05539-3. 39223577 PMC11370282

[13] Park J, Dean LS, Heckl J, Gangcuangco LM, Pedro TK, Tallquist MD, et al. Low-density granulocytes display immature cells with enhanced NET formation in people living with HIV. Sci Rep. 2023;13:13282. 10.1038/s41598-023-40475-0. 37587169 PMC10432506

[14] de Bont CM, Boelens WC, Pruijn GJM. NETosis, complement, and coagulation: a triangular relationship. Cell Mol Immunol. 2019;16:19–27. 10.1038/s41423-018-0024-0. 29572545 PMC6318284

[15] Li J, Chen J, Sun J, Li K. The formation of NETs and their mechanism of promoting tumor metastasis. J Oncol. 2023;2023:1–8. 10.1155/2023/7022337. PMC10024627 36942262

[16] Wu X, You D, Pan M, Weng M, Xie Q, Guan Y, et al. Knockout of the C3a receptor protects against renal ischemia reperfusion injury by reduction of NETs formation. Cell Mol Life Sci. 2023;80:322. 10.1007/s00018-023-04967-6. 37816851 PMC11072185

[17] Hirao H, Kojima H, Dery KJ, Nakamura K, Kadono K, Zhai Y, et al. Neutrophil CEACAM1 determines susceptibility to NETosis by regulating the S1PR2/S1PR3 axis in liver transplantation. J Clin Invest. 2023;133:e162940. 10.1172/JCI162940. 36719377 PMC9888387

[18] Kayhan M, Vouillamoz J, Rodriguez DG, Bugarski M, Mitamura Y, Gschwend J, et al. Intrinsic TGF-beta signaling attenuates proximal tubule mitochondrial injury and inflammation in chronic kidney disease. Nat Commun. 2023;14:3236. 10.1038/s41467-023-39050-y. 37270534 PMC10239443

[19] Fu Y, Wang W, Gong N, Zheng X, Guo X, Zhuang K, et al. Neutrophil and neutrophil extracellular traps in acute kidney injury: from mechanisms to treatments. Front Immunol. 2025;16:1688207. 10.3389/fimmu.2025.1688207. 41169399 PMC12568571

[20] Wang H, Kim SJ, Lei Y, Wang S, Wang H, Huang H, et al. Neutrophil extracellular traps in homeostasis and disease. Signal Transduct Target Ther. 2024;9:235. 10.1038/s41392-024-01933-x. 39300084 PMC11415080

[21] Menezes GB, Lee WY, Zhou H, Waterhouse CCM, Cara DC, Kubes P. Selective down-regulation of neutrophil Mac-1 in endotoxemic hepatic microcirculation via IL-10. J Immunol. 2009;183:7557–68. 10.4049/jimmunol.0901786. 19917697

[22] Li Y, Xu X, Wang HJ, Chen YC, Chen Y, Chiu J, et al. Endoplasmic reticulum protein 72 regulates integrin Mac-1 activity to influence neutrophil recruitment. Arterioscler Thromb Vasc Biol. 2024;44:e82–98. 10.1161/ATVBAHA.123.319771. 38205640

[23] Pang X, He X, Qiu Z, Zhang H, Xie R, Liu Z, et al. Targeting integrin pathways: mechanisms and advances in therapy. Signal Transduct Target Ther. 2023;8:1. 10.1038/s41392-022-01259-6. 36588107 PMC9805914

[24] Worthen GS, Smedly LA, Tonnesen MG, Ellis D, Voelkel NF, Reeves JT, et al. Effects of shear stress on adhesive interaction between neutrophils and cultured endothelial cells. J Appl Physiol. 1987;63:2031–41. 10.1152/jappl.1987.63.5.2031. 3693234

[25] Oliveira THC, Marques PE, Proost P, Teixeira MMM. Neutrophils: a cornerstone of liver ischemia and reperfusion injury. Lab Investig. 2018;98:51–62. 10.1038/labinvest.2017.90. 28920945

[26] Chackerian AA, Oldham ER, Murphy EE, Schmitz J, Pflanz S, Kastelein RA. IL-1 receptor accessory protein and ST2 comprise the IL-33 receptor complex. J Immunol. 2007;179:2551–5. 10.4049/jimmunol.179.4.2551. 17675517

[27] Li L, Wang H, Zhang J, Chen X, Zhang Z, Li Q. Effect of endothelial progenitor cell-derived extracellular vesicles on endothelial cell ferroptosis and atherosclerotic vascular endothelial injury. Cell Death Discov. 2021;7:235. 10.1038/s41420-021-00610-0. 34493702 PMC8423825

[28] Akbar N, Braithwaite AT, Corr EM, Koelwyn GJ, van Solingen C, Cochain C, et al. Rapid neutrophil mobilization by VCAM-1+ endothelial cell-derived extracellular vesicles. Cardiovasc Res. 2023;119:236–51. 10.1093/cvr/cvac012. 35134856 PMC10022859

[29] Nolan S, Dixon R, Norman K, Hellewell P, Ridger V. Nitric oxide regulates neutrophil migration through microparticle formation. Am J Pathol. 2008;172:265–73. 10.2353/ajpath.2008.070069. 18079439 PMC2189628

[30] Ge L, Zhou X, Ji WJ, Lu RY, Zhang Y, Zhang YD, et al. Neutrophil extracellular traps in ischemia-reperfusion injury-induced myocardial no-reflow: therapeutic potential of DNase-based reperfusion strategy. Am J Physiol Heart Circ Physiol. 2015;308:H500–9. 10.1152/ajpheart.00381.2014. 25527775

[31] Ma Y, Zabell T, Creasy A, Yang X, Chatterjee V, Villalba N, et al. Gut ischemia reperfusion injury induces lung inflammation via mesenteric lymph-mediated neutrophil activation. Front Immunol. 2020;11:586685. 10.3389/fimmu.2020.586685. 33042165 PMC7517702

[32] Dutta A, Das M, Ghosh A, Rana S. Molecular and cellular pathophysiology of circulating cardiomyocyte-specific cell free DNA (cfDNA): biomarkers of heart failure and potential therapeutic targets. Genes Dis. 2023;10:948–59. 10.1016/j.gendis.2022.08.008. 37396513 PMC10308167

[33] Vinten-Johansen J . Involvement of neutrophils in the pathogenesis of lethal myocardial reperfusion injury. Cardiovasc Res. 2004;61:481–97. 10.1016/j.cardiores.2003.10.011. 14962479

[34] Vafadarnejad E, Rizzo G, Krampert L, Arampatzi P, Arias-Loza AP, Nazzal Y, et al. Dynamics of cardiac neutrophil diversity in murine myocardial infarction. Circ Res. 2020;127:e232–49. 10.1161/CIRCRESAHA.120.317200. 32811295

[35] Zhang Y, Li X, Dai Y, Han Y, Wei X, Wei G, et al. Neutrophil N1 polarization induced by cardiomyocyte-derived extracellular vesicle miR-9-5p aggravates myocardial ischemia/reperfusion injury. J Nanobiotechnology. 2024;22:632. 10.1186/s12951-024-02902-w. 39415256 PMC11484374

[36] Tian H, Xiong Y, Zhan J, Lu Z, Zhang Y, Leng Y, et al. Inhibition of macrophage ARID3A alleviates myocardial ischemia-reperfusion injury after heart transplantation by reducing THBS1/CD47 signaling-mediated neutrophil extracellular traps formation. Adv Sci (Weinh). 2025;12:e09952. 10.1002/advs.202509952. 40913516 PMC12667509

[37] Gong Y, Lin J, Ma Z, Yu M, Wang M, Lai D, et al. Mitochondria-associated membrane-modulated Ca(2+) transfer: a potential treatment target in cardiac ischemia reperfusion injury and heart failure. Life Sci. 2021;278:119511. 10.1016/j.lfs.2021.119511. 33864818

[38] Missiroli S, Patergnani S, Caroccia N, Pedriali G, Perrone M, Previati M, et al. Mitochondria-associated membranes (MAMs) and inflammation. Cell Death Dis. 2018;9:329. 10.1038/s41419-017-0027-2. 29491386 PMC5832426

[39] Puente BN, Sun J, Parks RJ, Fergusson MM, Liu C, Springer DA, et al. MICU3 plays an important role in cardiovascular function. Circ Res. 2020;127:1571–3. 10.1161/CIRCRESAHA.120.317177. 33059536 PMC10809723

[40] Zhang H, Liu L, Shen C, Jiang X, Liu J, Chen J, et al. Lactate-induced mitochondrial calcium uptake 3 aggravates myocardial ischemia-reperfusion injury by promoting neutrophil extracellular trap formation. Research (Wash D C). 2025;8:0705. 10.34133/research.0705. 40452820 PMC12123085

[41] Fan Y, Lu J, Yu Z, Qu X, Guan S. 1,3-dichloro-2-propanol-induced renal tubular cell necroptosis through the ROS/RIPK3/MLKL pathway. J Agric Food Chem. 2022;70:10847–57. 10.1021/acs.jafc.2c02619. 36000575

[42] Chen H, Fang Y, Wu J, Chen H, Zou Z, Zhang X, et al. RIPK3-MLKL-mediated necroinflammation contributes to AKI progression to CKD. Cell Death Dis. 2018;9:878. 10.1038/s41419-018-0936-8. 30158627 PMC6115414

[43] Mulay SR, Linkermann A, Anders HJ. Necroinflammation in kidney disease. J Am Soc Nephrol. 2016;27:27–39. 10.1681/ASN.2015040405. 26334031 PMC4696588

[44] Boero E, Gorham RD, Jr, Francis EA, Brand J, Teng LH, Doorduijn DJ, et al. Purified complement C3b triggers phagocytosis and activation of human neutrophils via complement receptor 1. Sci Rep. 2023;13:274. 10.1038/s41598-022-27279-4. 36609665 PMC9822988

[45] Qi R, Qin W. Role of complement system in kidney transplantation: stepping from animal models to clinical application. Front Immunol. 2022;13:811696. 10.3389/fimmu.2022.811696. 35281019 PMC8913494

[46] Wu C, Xu J, Zhang Z, Wei D, Xu Y, Zhao Y. The effects of IL-23/IL-18-polarized neutrophils on renal ischemia-reperfusion injury and allogeneic-skin-graft rejection in mice. Biomedicines. 2023;11:3148. 10.3390/biomedicines11123148. 38137369 PMC10740676

[47] Dar WA, Sullivan E, Bynon JS, Eltzschig H, Ju C. Ischaemia reperfusion injury in liver transplantation: cellular and molecular mechanisms. Liver Int. 2019;39:788–801. 10.1111/liv.14091. 30843314 PMC6483869

[48] Kaltenmeier C, Yazdani HO, Handu S, Popp B, Geller D, Tohme S. The role of neutrophils as a driver in hepatic ischemia-reperfusion injury and cancer growth. Front Immunol. 2022;13:887565. 10.3389/fimmu.2022.887565. 35844608 PMC9284204

[49] Jaeschke H, Ramachandran A. Reactive oxygen species in the normal and acutely injured liver. J Hepatol. 2011;55:227–8. 10.1016/j.jhep.2011.01.006. 21238521 PMC3117914

[50] Turnage RH, Kadesky KM, Bartula L, Guice KS, Oldham KT, Myers SI. Splanchnic PGI2 release and ‘no reflow’ following intestinal reperfusion. J Surg Res. 1995;58:558–64. 10.1006/jsre.1995.1088. 7791328

[51] Liu Y, Pu X, Qin X, Gong J, Huang Z, Luo Y, et al. Neutrophil extracellular traps regulate HMGB1 translocation and Kupffer cell M1 polarization during acute liver transplantation rejection. Front Immunol. 2022;13:823511. 10.3389/fimmu.2022.823511. 35603144 PMC9120840

[52] Kato A, Edwards MJ, Lentsch AB. Gene deletion of NF-kappa B p50 does not alter the hepatic inflammatory response to ischemia/reperfusion. J Hepatol. 2002;37:48–55. 10.1016/S0168-8278(02)00068-5. 12076861

[53] Takahashi Y, Ganster RW, Gambotto A, Shao L, Kaizu T, Wu T, et al. Role of NF-kappaB on liver cold ischemia-reperfusion injury. Am J Physiol Gastrointest Liver Physiol. 2002;283:G1175–84. 10.1152/ajpgi.00515.2001. 12381532

[54] Kuboki S, Okaya T, Schuster R, Blanchard J, Denenberg A, Wong HR, et al. Hepatocyte NF-kappaB activation is hepatoprotective during ischemia-reperfusion injury and is augmented by ischemic hypothermia. Am J Physiol Gastrointest Liver Physiol. 2007;292:G201–7. 10.1152/ajpgi.00186.2006. 16950761

[55] Liu Y, Lei Z, Chai H, Kang Q, Qin X. Salidroside alleviates hepatic ischemia-reperfusion injury during liver transplant in rat through regulating TLR-4/NF-kappaB/NLRP3 inflammatory pathway. Sci Rep. 2022;12:13973. 10.1038/s41598-022-18369-4. 35978104 PMC9385636

[56] Liu Y, Lei Z, Chai H, Xiang S, Wang Y, Yan P, et al. Thrombomodulin-mediated inhibition of neutrophil extracellular trap formation alleviates hepatic ischemia-reperfusion injury by blocking TLR4 in rats subjected to liver transplantation. Transplantation. 2022;106:e126–40. 10.1097/TP.0000000000003954. 34534191

[57] Tara A, Dominic JL, Patel JN, Garg I, Yeon J, Memon MS, et al. Mitochondrial targeting therapy role in liver transplant preservation lines: mechanism and therapeutic strategies. Cureus. 2021;13:e16599. 10.7759/cureus.16599. 34430181 PMC8378417

[58] Zhang T, Liu P, Shen W, Li C, Zhao Z, Wu Y, et al. DNase I-mediated chemotactic nanoparticles for NETs targeting and microenvironment remodeling treatment of acute ischemic stroke. Adv Sci (Weinh). 2025;12:e03689. 10.1002/advs.202503689. 40536328 PMC12442667

[59] Kang L, Yu H, Yang X, Zhu Y, Bai X, Wang R, et al. Neutrophil extracellular traps released by neutrophils impair revascularization and vascular remodeling after stroke. Nat Commun. 2020;11:2488. 10.1038/s41467-020-16191-y. 32427863 PMC7237502

[60] Jin H, Li Z, Tan S, Xiao Q, Li Q, Ye J, et al. Neutrophil mobilization triggers microglial functional change to exacerbate cerebral ischemia-reperfusion injury. Adv Sci (Weinh). 2025;12:e03722. 10.1002/advs.202503722. 40557450 PMC12462934

[61] Oh SA, Seol SI, Davaanyam D, Kim SW, Lee JK. Platelet-derived HMGB1 induces NETosis, exacerbating brain damage in the photothrombotic stroke model. Mol Med. 2025;31:46. 10.1186/s10020-025-01107-7. 39910417 PMC11796003

[62] Chen J, Quan X, Li Y, Chen J, Hu J, Zhou M, et al. Siegesbeckia orientalis ethanol extract impedes RAGE-CD11b interaction driven by HMGB1 to alleviate neutrophil-involved neuronal injury poststroke. Phytomedicine. 2025;139:156541. 10.1016/j.phymed.2025.156541. 39986221

[63] Liu Z, Yang W, Chen J, Wang Q. Circulating HMGB1 in acute ischemic stroke and its association with post-stroke cognitive impairment. PeerJ. 2024;12:e17309. 10.7717/peerj.17309. 38708343 PMC11067911

[64] Zhang J, Miao C, Zhang H. Targeting neutrophil extracellular traps in cancer progression and metastasis. Theranostics. 2025;15:5846–69. 10.7150/thno.111096. 40365275 PMC12068306

[65] Yun H, Chi Y, Wei B, Bai H, Cao W, Zhang Z, et al. Cl-amidine confers organ protection and improves survival in hemorrhagic shock rats via the PAD4-CitH3-NETs axis. PLoS One. 2025;20:e0327085. 10.1371/journal.pone.0327085. 40591569 PMC12212502

[66] Shi Y, Liu T, Nieman DC, Cui Y, Li F, Yang L, et al. Aerobic exercise attenuates acute lung injury through NET inhibition. Front Immunol. 2020;11:409. 10.3389/fimmu.2020.00409. 32265910 PMC7096358

[67] Liu S, Su X, Pan P, Zhang L, Hu Y, Tan H, et al. Neutrophil extracellular traps are indirectly triggered by lipopolysaccharide and contribute to acute lung injury. Sci Rep. 2016;6:37252. 10.1038/srep37252. 27849031 PMC5110961

[68] Sha HX, Liu YB, Qiu YL, Zhong WJ, Yang NSY, Zhang CY, et al. Neutrophil extracellular traps trigger alveolar epithelial cell necroptosis through the cGAS-STING pathway during acute lung injury in mice. Int J Biol Sci. 2024;20:4713–30. 10.7150/ijbs.99456. 39309425 PMC11414388

[69] Zhu S, Yu Y, Qu M, Qiu Z, Zhang H, Miao C, et al. Neutrophil extracellular traps contribute to immunothrombosis formation via the STING pathway in sepsis-associated lung injury. Cell Death Discov. 2023;9:315. 10.1038/s41420-023-01614-8. 37626060 PMC10457383

[70] Wu J, Gao P, Yang C, Zhuang F, Luo Y, Wen F, et al. Targeting mitochondrial complex I of CD177+ neutrophils alleviates lung ischemia-reperfusion injury. Cell Rep Med. 2025;6:102140. 10.1016/j.xcrm.2025.102140. 40398393 PMC12147905

[71] Zeng L, Xiang W, Xiao W, Wu Y, Sun L. The emerging role of neutrophil extracellular traps in autoimmune and autoinflammatory diseases. MedComm. 2020;6:e70101. 10.1002/mco2.70101. PMC11885892 40060194

[72] Chu C, Wang X, Chen F, Yang C, Shi L, Xu W, et al. Neutrophil extracellular traps aggravate intestinal epithelial necroptosis in ischaemia-reperfusion by regulating TLR4/RIPK3/FUNDC1-required mitophagy. Cell Prolif. 2024;57:e13538. 10.1111/cpr.13538. 37691112 PMC10771116

[73] Tang J, Yue J, Tao Y, Zhao G, Yi X, Zhang M, et al. Neutrophil extracellular traps induce brain edema around intracerebral hematoma via ERK-mediated regulation of MMP9 and AQP4. Transl Stroke Res. 2025;16:1461–73. 10.1007/s12975-024-01318-w. 39733198 PMC12391242

[74] Middleton EA, He XY, Denorme F, Campbell RA, Ng D, Salvatore SP, et al. Neutrophil extracellular traps contribute to immunothrombosis in COVID-19 acute respiratory distress syndrome. Blood. 2020;136:1169–79. 10.1182/blood.2020007008. 32597954 PMC7472714

[75] Chu C, Wang X, Yang C, Chen F, Shi L, Xu W, et al. Neutrophil extracellular traps drive intestinal microvascular endothelial ferroptosis by impairing Fundc1-dependent mitophagy. Redox Biol. 2023;67:102906. 10.1016/j.redox.2023.102906. 37812880 PMC10579540

[76] Zhou T, Prather ER, Garrison DE, Zuo L. Interplay between ROS and antioxidants during ischemia-reperfusion injuries in cardiac and skeletal muscle. Int J Mol Sci. 2018;19:417. 10.3390/ijms19020417. 29385043 PMC5855639

[77] Zhang F, Li Y, Wu J, Zhang J, Cao P, Sun Z, et al. The role of extracellular traps in ischemia reperfusion injury. Front Immunol. 2022;13:1022380. 10.3389/fimmu.2022.1022380. 36211432 PMC9533173

[78] Dugbartey GJ . Cellular and molecular mechanisms of cell damage and cell death in ischemia-reperfusion injury in organ transplantation. Mol Biol Rep. 2024;51:473. 10.1007/s11033-024-09261-7. 38553658 PMC10980643

[79] Zhuang S, Xia S, Huang P, Wu J, Qu J, Chen R, et al. Targeting P2RX1 alleviates renal ischemia/reperfusion injury by preserving mitochondrial dynamics. Pharmacol Res. 2021;170:105712. 10.1016/j.phrs.2021.105712. 34091010

[80] Nakazawa D, Kumar SV, Marschner J, Desai J, Holderied A, Rath L, et al. Histones and Neutrophil Extracellular Traps Enhance Tubular Necrosis and Remote Organ Injury in Ischemic AKI. J Am Soc Nephrol. 2017;28:1753–68. 10.1681/asn.2016080925. PMC5461800 28073931

[81] Gao J, Zhang Z, Yu J, Zhang N, Fu Y, Jiang X, et al. Identification of neutrophil extracellular trap-related gene expression signatures in ischemia reperfusion injury during lung transplantation: a transcriptome analysis and clinical validation. J Inflamm Res. 2024;17:981–1001. 10.2147/JIR.S444774. 38370470 PMC10871139

[82] Hayase N, Doi K, Hiruma T, Matsuura R, Hamasaki Y, Noiri E, et al. Recombinant thrombomodulin on neutrophil extracellular traps in murine intestinal ischemia-reperfusion. Anesthesiology. 2019;131:866–82. 10.1097/ALN.0000000000002898. 31453815

[83] Harada T, Shimomura Y, Nishida O, Maeda M, Kato Y, Nakamura T, et al. Effects of recombinant human soluble thrombomodulin on neutrophil extracellular traps in the kidney of a mouse model of endotoxin shock. Fujita Med J. 2023;9:225–30. 10.20407/fmj.2022-026. 37554943 PMC10405902

[84] Shen Y, You Q, Wu Y, Wu J. Inhibition of PAD4-mediated NET formation by cl-amidine prevents diabetes development in nonobese diabetic mice. Eur J Pharmacol. 2022;916:174623. 10.1016/j.ejphar.2021.174623. 34767782

[85] Grayson PC, Kaplan MJ. At the bench: neutrophil extracellular traps (NETs) highlight novel aspects of innate immune system involvement in autoimmune diseases. J Leukoc Biol. 2016;99:253–64. 10.1189/jlb.5BT0615-247R. 26432901 PMC4718195

[86] Mu Q, Yao K, Syeda MZ, Wan J, Cheng Q, You Z, et al. Neutrophil targeting platform reduces neutrophil extracellular traps for improved traumatic brain injury and stroke theranostics. Adv Sci (Weinh). 2024;11:e2308719. 10.1002/advs.202308719. 38520727 PMC11151022

[87] Yang K, Gao R, Chen H, Hu J, Zhang P, Wei X, et al. Myocardial reperfusion injury exacerbation due to ALDH2 deficiency is mediated by neutrophil extracellular traps and prevented by leukotriene C4 inhibition. Eur Heart J. 2024;45:1662–80. 10.1093/eurheartj/ehae205. 38666340 PMC11089336

[88] Jia M, Miao W, Li Y, Guo Y, Zeng J, Gao Y, et al. A polymerized probucol nanoformulation with neutrophil extracellular vesicle camouflage for cerebral ischemia-reperfusion injury therapy. Innovation (Camb). 2025;6:100761. 10.1016/j.xinn.2024.100761. 40470331 PMC12131010

[89] Hu S, Zhang F, Wang J, Zhang J, Li C, Lyu Y, et al. MMP9(high) neutrophils are critical mediators of neutrophil extracellular traps formation and myocardial ischemia/reperfusion injury. Adv Sci (Weinh). 2025;12:e2415205. 10.1002/advs.202415205. 40151877 PMC12140383

[90] Yuan ZL, Mo YZ, Li DL, Xie L, Chen MH. Inhibition of ERK downregulates autophagy via mitigating mitochondrial fragmentation to protect SH-SY5Y cells from OGD/R injury. Cell Commun Signal. 2023;21:204. 10.1186/s12964-023-01211-3. 37580749 PMC10426156

[91] Burgener SS, Schroder K. Neutrophil extracellular traps in host defense. Cold Spring Harb Perspect Biol. 2020;12:a037028. 10.1101/cshperspect.a037028. 31767647 PMC7328462

[92] Demkow U . Molecular mechanisms of neutrophil extracellular trap (NETs) degradation. Int J Mol Sci. 2023;24:4896. 10.3390/ijms24054896. 36902325 PMC10002918

[93] Arumugam TV, Woodruff TM, Stocks SZ, Proctor LM, Pollitt S, Shiels IA, et al. Protective effect of a human C5a receptor antagonist against hepatic ischaemia-reperfusion injury in rats. J Hepatol. 2004;40:934–41. 10.1016/j.jhep.2004.02.017. 15158333

[94] Chen Y, Li X, Lin X, Liang H, Liu X, Zhang X, et al. Complement C5a induces the generation of neutrophil extracellular traps by inhibiting mitochondrial STAT3 to promote the development of arterial thrombosis. Thromb J. 2022;20:24. 10.1186/s12959-022-00384-0. 35488279 PMC9051782

[95] Huang YM, Wang H, Wang C, Chen M, Zhao MH. Promotion of hypercoagulability in antineutrophil cytoplasmic antibody-associated vasculitis by C5a-induced tissue factor-expressing microparticles and neutrophil extracellular traps. Arthritis Rheumatol. 2015;67:2780–90. 10.1002/art.39239. 26097236

[96] Jayne DRW, Merkel PA, Schall TJ, Bekker P. Avacopan for the treatment of ANCA-associated vasculitis. N Engl J Med. 2021;384:599–609. 10.1056/NEJMoa2023386. 33596356

[97] Harigai M, Takada H. Avacopan, a selective C5a receptor antagonist, for anti-neutrophil cytoplasmic antibody-associated vasculitis. Mod Rheumatol. 2022;32:475–83. 10.1093/mr/roab104. 34984461

[98] Ahmad A, Mandwie M, O'Sullivan KM, Smyth C, York J, Doyle H, et al. Conversion of the liver into a biofactory for DNaseI using adeno-associated virus vector gene transfer reduces neutrophil extracellular traps in a model of systemic lupus erythematosus. Hum Gene Ther. 2022;33:560–71. 10.1089/hum.2021.264. 35293226

[99] Silachev DN, Isaev NK, Pevzner IB, Zorova LD, Stelmashook EV, Novikova SV, et al. The mitochondria-targeted antioxidants and remote kidney preconditioning ameliorate brain damage through kidney-to-brain cross-talk. PLoS One. 2012;7:e51553. 10.1371/journal.pone.0051553. 23272118 PMC3522699

[100] Andreev-Andrievskiy AA, Kolosova NG, Stefanova NA, Lovat MV, Egorov MV, Manskikh VN, et al. Efficacy of mitochondrial antioxidant plastoquinonyl-decyl-triphenylphosphonium bromide (SkQ1) in the rat model of autoimmune arthritis. Oxidative Med Cell Longev. 2016;2016:8703645. 10.1155/2016/8703645. PMC4887630 27293517

[101] Jia B, Ye J, Gan L, Li R, Zhang M, Sun D, et al. Mitochondrial antioxidant SkQ1 decreases inflammation following hemorrhagic shock by protecting myocardial mitochondria. Front Physiol. 2022;13:1047909. 10.3389/fphys.2022.1047909. 36467681 PMC9709459

[102] Zhu D, Lu Y, Yang S, Hu T, Tan C, Liang R, et al. PAD4 inhibitor-functionalized layered double hydroxide nanosheets for synergistic sonodynamic therapy/immunotherapy of tumor metastasis. Adv Sci (Weinh). 2024;11:e2401064. 10.1002/advs.202401064. 38708711 PMC11234469

[103] Liu X, Li T, Chen H, Yuan L, Ao H. Role and intervention of PAD4 in NETs in acute respiratory distress syndrome. Respir Res. 2024;25:63. 10.1186/s12931-024-02676-7. 38291476 PMC10829387

[104] Uysal-Onganer P, D'Alessio S, Mortoglou M, Kraev I, Lange S. Peptidylarginine deiminase inhibitor application, using Cl-amidine, PAD2, PAD3 and PAD4 isozyme-specific inhibitors in pancreatic cancer cells, reveals roles for PAD2 and PAD3 in cancer invasion and modulation of extracellular vesicle signatures. Int J Mol Sci. 2021;22:1396. 10.3390/ijms22031396. 33573274 PMC7866560

[105] Deng H, Lin C, Garcia-Gerique L, Fu S, Cruz Z, Bonner EE, et al. A novel selective inhibitor JBI-589 targets PAD4-mediated neutrophil migration to suppress tumor progression. Cancer Res. 2022;82:3561–72. 10.1158/0008-5472.CAN-21-4045. 36069973 PMC9532374

[106] Li H, Li C, Fu C, Wang Y, Liang T, Wu H, et al. Innovative nanoparticle-based approaches for modulating neutrophil extracellular traps in diseases: from mechanisms to therapeutics. J Nanobiotechnology. 2025;23:88. 10.1186/s12951-025-03195-3. 39915767 PMC11800495

[107] Morand EF, Furie R, Tanaka Y, Bruce IN, Askanase AD, Richez C, et al. Trial of anifrolumab in active systemic lupus erythematosus. N Engl J Med. 2020;382:211–21. 10.1056/NEJMoa1912196. 31851795

[108] Furumoto Y, Smith CK, Blanco L, Zhao W, Brooks SR, Thacker SG, et al. Tofacitinib ameliorates murine lupus and its associated vascular dysfunction. Arthritis Rheumatol. 2017;69:148–60. 10.1002/art.39818. 27429362 PMC5195893

[109] Hasni SA, Gupta S, Davis M, Poncio E, Temesgen-Oyelakin Y, Carlucci PM, et al. Phase 1 double-blind randomized safety trial of the Janus kinase inhibitor tofacitinib in systemic lupus erythematosus. Nat Commun. 2021;12:3391. 10.1038/s41467-021-23361-z. 34099646 PMC8185103

[110] Shak S . Aerosolized recombinant human DNase I for the treatment of cystic fibrosis. Chest. 1995;107:65S–70. 10.1378/chest.107.2_Supplement.65S. 7842816

[111] Wang CL, Wang Y, Jiang QL, Zeng Y, Yao QP, Liu X, et al. DNase I and sivelestat ameliorate experimental hindlimb ischemia-reperfusion injury by eliminating neutrophil extracellular traps. J Inflamm Res. 2023;16:707–21. 10.2147/JIR.S396049. 36852300 PMC9961174

[112] Jarrahi A, Khodadadi H, Moore NS, Lu Y, Awad ME, Salles EL, et al. Recombinant human DNase-I improves acute respiratory distress syndrome via neutrophil extracellular trap degradation. J Thromb Haemost. 2023;21:2473–84. 10.1016/j.jtha.2023.04.044. 37196848 PMC10185489

[113] Ma W, Wu D, Long C, Liu J, Xu L, Zhou L, et al. Neutrophil-derived nanovesicles deliver IL-37 to mitigate renal ischemia-reperfusion injury via endothelial cell targeting. J Control Release. 2024;370:66–81. 10.1016/j.jconrel.2024.04.025. 38631490

[114] Keir HR, Shoemark A, Dicker AJ, Perea L, Pollock J, Giam YH, et al. Neutrophil extracellular traps, disease severity, and antibiotic response in bronchiectasis: an international, observational, multicohort study. Lancet Respir Med. 2021;9:873–84. 10.1016/S2213-2600(20)30504-X. 33609487

[115] Hoeben D, Dosogne H, Heyneman R, Burvenich C. Effect of antibiotics on the phagocytotic and respiratory burst activity of bovine granulocytes. Eur J Pharmacol. 1997;332:289–97. 10.1016/S0014-2999(97)01107-2. 9300263

[116] Manda-Handzlik A, Bystrzycka W, Sieczkowska S, Demkow U, Ciepiela O. Antibiotics modulate the ability of neutrophils to release neutrophil extracellular traps. Adv Exp Med Biol. 2017;944:47–52. 10.1007/5584_2016_59. 27826884

[117] Behal ML, Nguyen JL, Li X, Feola DJ, Neyra JA, Flannery AH. Azithromycin and major adverse kidney events in critically ill patients with sepsis-associated acute kidney injury. Shock. 2022;57:479–85. 10.1097/SHK.0000000000001883. 34731096 PMC9725110

[118] Shrestha B, Ito T, Kakuuchi M, Totoki T, Nagasato T, Yamamoto M, et al. Recombinant thrombomodulin suppresses histone-induced neutrophil extracellular trap formation. Front Immunol. 2019;10:2535. 10.3389/fimmu.2019.02535. 31736962 PMC6828967

[119] Helms J, Clere-Jehl R, Bianchini E, le Borgne P, Burban M, Zobairi F, et al. Thrombomodulin favors leukocyte microvesicle fibrinolytic activity, reduces NETosis and prevents septic shock-induced coagulopathy in rats. Ann Intensive Care. 2017;7:118. 10.1186/s13613-017-0340-z. 29222696 PMC5722785

[120] Nakahara M, Ito T, Kawahara K, Yamamoto M, Nagasato T, Shrestha B, et al. Recombinant thrombomodulin protects mice against histone-induced lethal thromboembolism. PLoS One. 2013;8:e75961. 10.1371/journal.pone.0075961. 24098750 PMC3786915

